# Additive Effect on Survival of Anaesthetic Cardiac Protection and Remote Ischemic Preconditioning in Cardiac Surgery: A Bayesian Network Meta-Analysis of Randomized Trials

**DOI:** 10.1371/journal.pone.0134264

**Published:** 2015-07-31

**Authors:** Alberto Zangrillo, Mario Musu, Teresa Greco, Ambra Licia Di Prima, Andrea Matteazzi, Valentina Testa, Pasquale Nardelli, Daniela Febres, Fabrizio Monaco, Maria Grazia Calabrò, Jun Ma, Gabriele Finco, Giovanni Landoni

**Affiliations:** 1 Department of Anesthesia and Intensive Care, IRCCS San Raffaele Scientific Institute, Milan, Italy; 2 Vita-Salute San Raffaele University, Milan, Italy; 3 Department of Medical Sciences “M. Aresu”, Cagliari University, Cagliari, Italy; 4 Center for Anesthesiology, Beijing Anzhen Hospital, Capital Medical University, Beijing, China; University of Colorado Denver, UNITED STATES

## Abstract

**Introduction:**

Cardioprotective properties of volatile agents and of remote ischemic preconditioning have survival effects in patients undergoing cardiac surgery. We performed a Bayesian network meta-analysis to confirm the beneficial effects of these strategies on survival in cardiac surgery, to evaluate which is the best strategy and if these strategies have additive or competitive effects.

**Methods:**

Pertinent studies were independently searched in BioMedCentral, MEDLINE/PubMed, Embase, and the Cochrane Central Register (updated November 2013). A Bayesian network meta-analysis was performed. Four groups of patients were compared: total intravenous anesthesia (with or without remote ischemic preconditioning) and an anesthesia plan including volatile agents (with or without remote ischemic preconditioning). Mortality was the main investigated outcome.

**Results:**

We identified 55 randomized trials published between 1991 and 2013 and including 6,921 patients undergoing cardiac surgery. The use of volatile agents (posterior mean of odds ratio = 0.50, 95% CrI 0.28–0.91) and the combination of volatile agents with remote preconditioning (posterior mean of odds ratio = 0.15, 95% CrI 0.04–0.55) were associated with a reduction in mortality when compared to total intravenous anesthesia. Posterior distribution of the probability of each treatment to be the best one, showed that the association of volatile anesthetic and remote ischemic preconditioning is the best treatment to improve short- and long-term survival after cardiac surgery, suggesting an additive effect of these two strategies.

**Conclusions:**

In patients undergoing cardiac surgery, the use of volatile anesthetics and the combination of volatile agents with remote preconditioning reduce mortality when compared to TIVA and have additive effects. It is necessary to confirm these results with large, multicenter, randomized, double-blinded trials comparing these different strategies in cardiac and non-cardiac surgery, to establish which volatile agent is more protective than the others and how to best apply remote ischemic preconditioning.

## Introduction

Acute cardiac damage in cardiac surgery is associated with increased morbidity and mortality and the identification of cardioprotective strategies might influence patients’ outcome. [1] Cardioprotection refers to all interventions that can attenuate cardiac damage induced by myocardial ischemia and reperfusion.

Ischemic preconditioning is a response at cellular level to brief sub-lethal episodes of ischemia leading to a major protection against subsequent lethal ischemia. Remote ischemic preconditioning consists in the stimulation of brief episodes of ischemia and reperfusion in a tissue or in an organ different from the heart (e.g. limb ischemia), inducing myocardial protection from ischemic injury. [2] Recent meta-analyses [3,4] suggested that remote ischemic preconditioning significantly reduces postoperative cardiac biomarkers release in adult cardiac surgery and a RCT found a reduction in mortality in patients receiving remote ischemic preconditioning. [5]

Volatile agents are among the few interventions that might reduce perioperative mortality, [6,7] probably by reproducing anti-ischemic effects. Recent studies have shown that volatile agents can also mimic the early phase of ischemic preconditioning. [8]

To address the question whether the choice of anesthetic regimen and the application of remote ischemic preconditioning have beneficial additive effects on patients’ survival after cardiac surgery, we carried out a Bayesian network meta-analysis, a valid and robust statistical technique that compares indirectly various treatments.

## Methods

We performed a network Bayesian meta-analysis in order to evaluate the effects on mortality of remote preconditioning, volatile agents and total intravenous anesthesia (TIVA). Our aim was to assess whether remote preconditioning and the choice of anesthetic regimen can influence survival on patients after cardiac surgery.

### Search strategy and study selection

The search for relevant studies was carried out independently in BioMedCentral, Embase, MEDLINE/PubMed and in the Cochrane Central Register of clinical trials by two experienced investigators. Literature searches were last updated on November 1^st^ 2013. The full PubMed search strategy is available in the S1 File. [9]

All references obtained from literature searches with the abovementioned methods were independently evaluated at a title/abstract level by two investigators and then, if potentially reasonable, assessed as full-text articles. Non-English articles were translated in order not to exclude them in the analyses. Inclusion criteria were random allocation to treatment and comparison between a total intravenous anesthesia (TIVA) and a combined plan including administration of a volatile agent (isoflurane, desflurane or sevoflurane); comparison between the use of remote ischemic preconditioning and not; cardiac surgery setting. There was no restriction in dose and time of anesthetic administration or methods of remote ischemic preconditioning. The exclusion criteria were missing outcome data, experimental studies on animal subjects and duplicate publications. In case of duplicate publications we included the article reporting the longest follow-up. We also included in the analysis studies in which epidural anesthesia or analgesia was given to all subjects.

Two investigators were independently tasked with verifying study compatibility with selection criteria. Any potential divergences were sorted out by agreement.

### Data Abstraction and study characteristics

Two trained investigators independently obtained baseline, procedural and outcome data with divergences resolved by consensus (S1 Table).

Specifically, we extracted survival, study design (including patient selection, randomization, single-/multi-center design), clinical setting, population, and anesthetic comparators. “TIVA” was defined as a group not receiving desflurane, isoflurane or sevoflurane. “Volatile” was defined as a group receiving at least 5 minutes of a volatile agent (even if added over a TIVA regimen). “Remote” was defined as a group receiving remote ischemic preconditioning.

The endpoint of the present review was to identify the effects on mortality at the longest follow-up available of remote ischemic preconditioning, volatile agents and TIVA using a network Bayesian meta-analysis. If the study had missing or incomplete data on survival we contacted all authors by e-mail, letter or both.

### Internal Validity and Risk of Bias Assessment

The internal validity of each trial included in this review was carefully evaluated for bias in accordance with The Cochrane Collaboration guidelines. [10] We assessed the potential source of bias by applying ‘Yes’, ‘No’ or ‘Unclear’ to the Cochrane checklist and by expressing the overall risk of bias as ‘low’, ‘moderate’ or ‘high’.

The posterior probability check method [11] was used to test the consistency assumption, i.e. no discrepancy between direct and indirect comparisons. We compared the difference in residual deviance between the consistency model (which estimates the indirect treatment effects by consistency equation) [12] with the inconsistency one (which estimates all the relative effects for all the treatment contrasts). [13] We defined as ‘treatment effect’ the difference in the drug’s effect between two treatments.

### Statistical Analysis

For each trial, the number of reported deaths and number of patients included were expressed as the logarithm of odds ratio (OR) and of its standard error (SE). If available, we used data referred to the intention-to-treat population.

The primary treatment strategies of interest in this network meta-analysis were 1) TIVA, 2) volatile, 3) remote-TIVA and 4) remote-volatile. A graphical representation of the network was used to show the pairwise association between each treatment. In this diagram each node represents a single anesthetic agent and each edge connects treatments that have been directly compared in one or more RCTs. The font size of each anesthetic agent node is proportional to the number of randomized patients per group and the edge size is proportional to the number of studies per comparisons. The dashed line indicates the comparison derived from a multi-arm trial.

To analyze the binary outcome, among pairwise comparisons and through standard meta-analysis, we pooled the study-specific estimates using the inverse-variance method and a fixed-effect model. [10] To assess the presence of study heterogeneity, we used the Cochran's *Q* statistic and the I-square statistic (I-square >25% was used as a threshold indicating significant heterogeneity).

We performed the network analysis modeling the binary outcome for mortality with a Bayesian hierarchical model (binomial model with logit link function) using the Markov chain Monte Carlo (MCMC) estimating algorithm. We used non-informative priors to produce the posterior distributions for the treatment effect in reference group (TIVA) and the treatment difference effects (Normal distribution with mean equal to 0 and variance equal to 0.0001). To overcome the zero-cell count problem, we ran the random effect model with a more informative prior (Inverse-Gamma distribution) on the variance parameter. [13,14] Model results were based on 3 Markov chains: 50,000 interactions after a burn-in of 50,000 for the fixed models and 100,000 interactions after a burn-in of 100,000 for the random effect models. We compared the fit of the fixed or random effect model by calculating the posterior mean of residual deviance (D_res_) and the Deviance Information Criterion (DIC) statistics. We selected the random effect model because it is usually the most conservative option.

Pooled ORs, comparing different anesthetic agents, were estimated from the mean of the posterior distribution obtained with the Bayesian approach. After confirming the consistency hypothesis, the indirect estimate was calculated as difference from the appropriate direct estimates; the corresponding 95% credibility intervals (CrI) were obtained by normal approximation. We assumed that the between-trial variance is homogeneous across treatment contrast and we took into account the correlation between the treatment difference effect for each group of multi-arm trials. [15,16] We checked whether a model’s fit was satisfactory by examining the convergence of the MCMC simulation algorithm.

We performed two meta-regressions to evaluate the statistical influence on treatment effects. We included in the model (i) the length of follow-up and (ii) the study-specific precision (measured as inverse of sample size) to detect the potential publication bias.

Sensitivity meta-analyses were implemented analyzing data from studies with low risk of bias, with trials with more than 80 patients and removing the largest study of Hong DM et al. [17]

The statistical analysis will be performed by winBUGS (release 1.4, freeware available by BUGS project) and STATA (release 11, College Station, TX). Statistical significance was set at the two-tailed 0.05 level for hypothesis testing. Unadjusted P values are reported throughout.

This study was performed in compliance with The Cochrane Collaboration and Preferred Reporting Items for Systematic Reviews and Meta-Analyses guidelines (S1 Checklist). [10,18]

## Results

### Description of included trials

**Fig 1:**
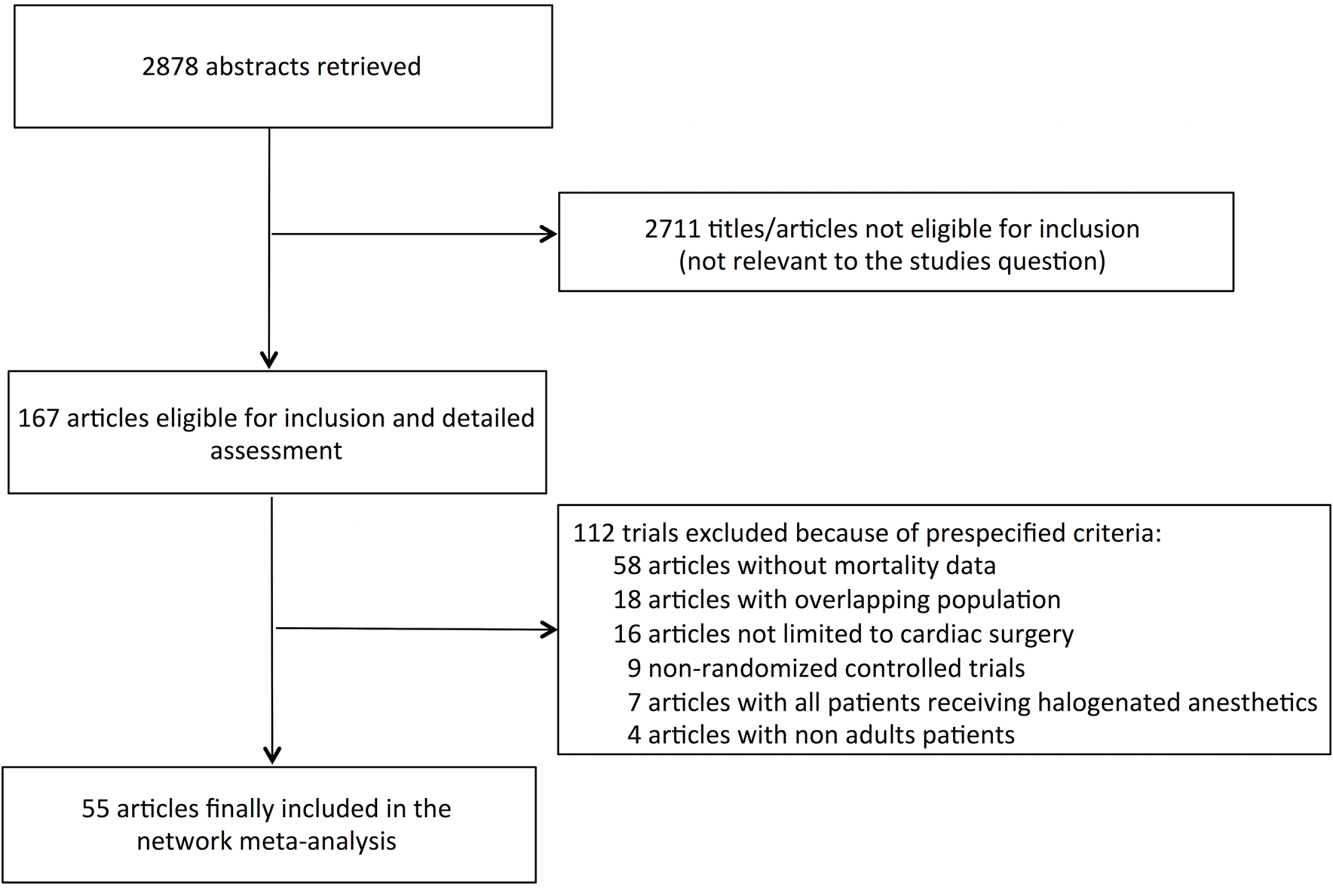
Flow diagram for selection of articles.

The process of selection of randomized controlled trials is shown in Fig 1. Our search strategy identified 2,878 unique publications, the titles and abstracts of which were screened for inclusion. The full text of 112 articles was retrieved. Among these, we excluded 58 studies since there were no outcome data and it was not possible to obtain further details by the authors, 18 studies due to overlapping populations, 16 articles performed in a setting that was not cardiac surgery, 9 non-randomized studies, 7 studies in which all patients received a halogenated administration and 4 pediatric trials (references of excluded trials available contacting the Authors).

We finally spotted 55 eligible randomized clinical trials, [5,15,16,17,19–69] which met the inclusion criteria and were comprised in the final analysis. S1 Table reports the clinical data on outcome, length of follow-up and type of comparator.

### Study Characteristics

The 55 included trials randomized 6,921 patients including 2,704 (39% in 50 studies) receiving volatile agents, 2,564 (37% in 41 studies) receiving TIVA, 902 (13% in 7 studies) receiving remote-TIVA, and 751 (11% in 15 studies) receiving remote-volatile. The most common pairwise comparison was volatile agents versus TIVA (34 [62%] studies) followed by volatile agents versus remote-volatile (14 [25%] studies) and TIVA versus remote-TIVA (5 [9.1%] studies). We included one three-arm study [15] and one four-arm study. [16] The dates of trial publication range between 1991 and 2013 and the trials randomized a median of 76 (48–124) patients.

Study quality appraisal indicated that studies were of medium/high quality (S2 Table) and that 30 [55%] of them had a low risk of bias.

### Quantitative Data Synthesis

**Fig 2:**
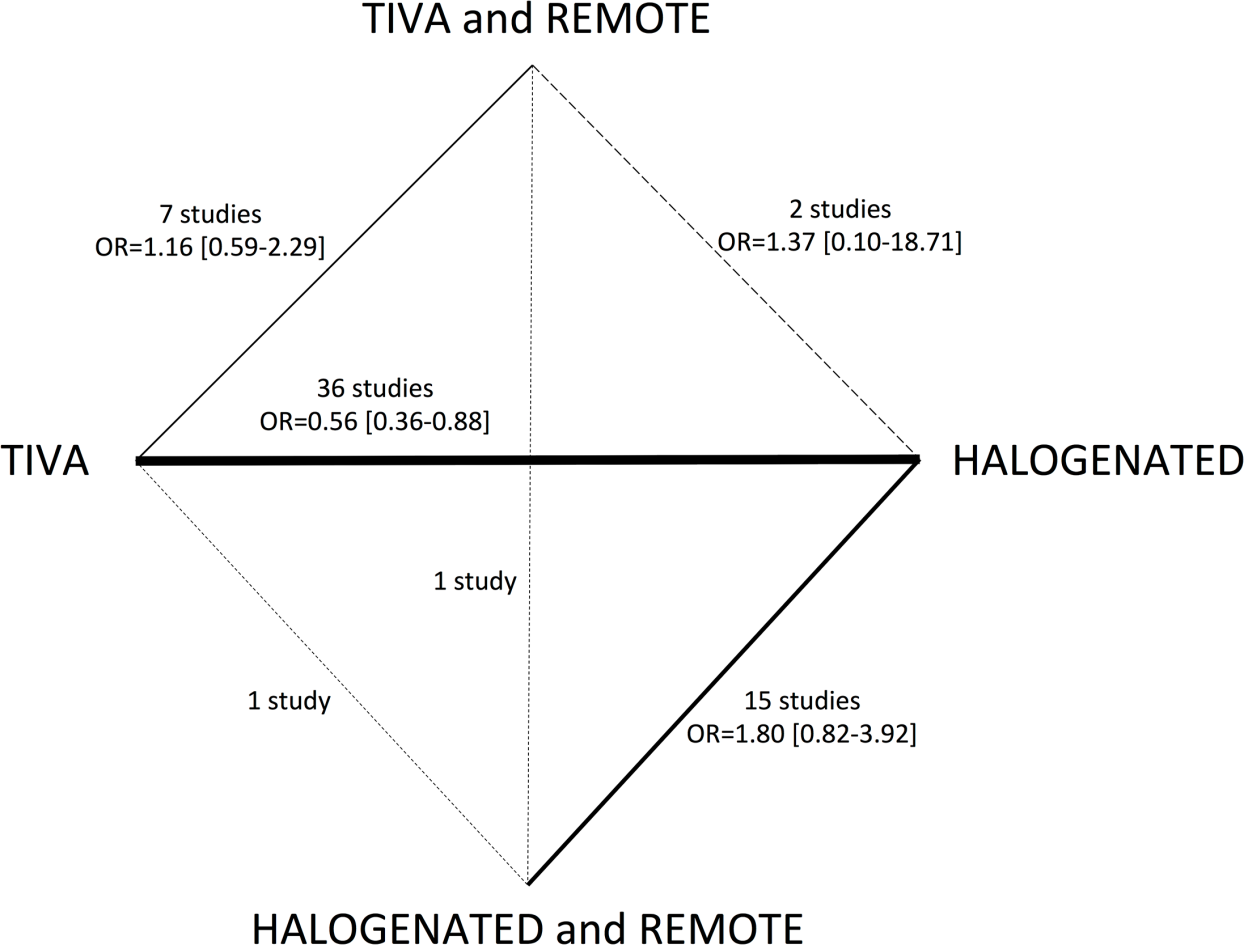
Network configuration. Comparisons between treatments and number of studies for each contrast.

The network configuration of each contrast analyzed by a Bayesian network meta-analysis is reported in Fig 2.

When comparing TIVA, volatile, remote-TIVA, remote-volatile through simple direct comparisons we found the following differences in mortality: 1) volatile agents (27/1997 [1.4%]) vs. TIVA (41/1683 [2.4%]), OR = 0.56, 95% IC 0.36–0.88, p for effect = 0.01, I-square = 0% with 36 studied included (S2 Fig); 2) volatile agents (15/726 [2.1%]) vs. remote-volatile (5/751 [0.7%]), OR = 1.80, 95% IC 0.82–3.92, p for effect = 0.14, I-square = 0% with 15 studied included (S3 Fig); 3) TIVA (17/915 [1.9%]) vs. remote-TIVA (14/902 [1.6%]), OR = 0.1.16, 95% IC 0.59–2.29, p for effect = 0.7, I-square = 0% with 7 studied included (S4 Fig); 4) volatile (1/34[2.9%]) vs. remote-TIVA (0/29), OR = 1.37, 95% IC 0.10–18.71, p for effect = 0.8, I-square = 0% with 2 studied included (S5 Fig).

The random (D_res_ = 159.6 and DIC = 188.7) and fixed (D_res_ = 160.6 and DIC = 188.5) effects models had no differences in model fit. To estimate the treatment effect, we selected the first that is the most conservative method. The WinBUGS code and the data entry, used to fit the random effect model, are available in the S2 File. The final results are reported in Table 1. The indirect estimate was calculated as difference from the appropriate direct estimates (probability in favor of inconsistency model equal to 0.03). We calculated the indirect 95% CrI by Normal approximation.

**Table 1:** Posterior distribution of mean and 95% credible interval, for the anesthetic agent difference effects, derived by Bayesian hierarchical model.

	Overall		Low-risk-of-bias studies	
Contrast	OR	95% credible interval	OR	95% credible interval
Volatile vs. TIVA	0.50 ^sign^	0.28–0.91	0.43 ^sign^	0.20–0.97
Remote-TIVA vs. TIVA	0.79	0.32–1.93	0.55	0.12–1.67
Remote-Volatile vs. TIVA	0.15 ^sign^	0.04–0.55	0.13 ^sign^	0.03–0.56
Volatile vs. Remote-TIVA *	0.63	0.22–1.85	0.79	0.16–3.85
Remote-Volatile vs. Volatile *	0.31	0.07–1.27	0.31	0.06–1.67
Remote-Volatile vs. Remote-TIVA *	0.19 ^sign^	0.04–0.94	0.25	0.03–1.88
	**Studies with more than 80 patients**		**Removing the largest study of Hong DM et al** ^**17**^	
**Contrast**	**OR**	**95% credible interval**	**OR**	**95% credible interval**
Volatile vs. TIVA	0.40 ^sign^	0.21–0.75	0.50 ^sign^	0.28–0.96
Remote-TIVA vs. TIVA	0.91	0.36–2.46	0.99	0.17–4.98
Remote-Volatile vs. TIVA	0.15 ^sign^	0.04–0.58	0.16 ^sign^	0.04–0.59
Volatile vs. Remote-TIVA *	0.44	0.14–1.39	0.50	0.08–3.03
Remote-Volatile vs. Volatile *	0.37	0.08–1.75	0.32	0.07–1.41
Remote-Volatile vs. Remote-TIVA *	0.16 ^sign^	0.03–0.89	0.16	0.02–1.39

* Indirect treatment difference effect calculated from consistency equation.

^sign^ Significant treatment difference effect.

TIVA (Total intravenous anesthesia) is the reference treatment.

TIVA: Total intravenous anesthesia

The Bayesian network meta-analysis found that the use of volatile agents (posterior mean of OR = 0.50, 95% CrI 0.28–0.91) and remote-volatile (posterior mean of OR = 0.15, 95% CrI 0.04–0.55) were associated with decreased mortality when compared to TIVA at the longest follow-up available. Besides, we observed that the use of remote-volatile was as well associated with a reduction in mortality when compared to remote-TIVA (posterior mean of OR = 0.19, 95% CrI 0.04–0.94).

Different length of follow-up did not affect our findings as documented by the ORs that were constant over time: posterior mean of slope coefficient = 0.00003, 95% CrI -0.004 to 0.005. Furthermore, the study precision was not associated with the risk of mortality (posterior mean of slope coefficient = 34.3, 95% CrI -50.8 to 118.5).

When repeating the analysis using low-risk-of-bias studies, we confirmed the significant reduction in mortality using halogenated (posterior mean of OR = 0.43, 95% CrI 0.20–0.97) and remote-volatile (posterior mean of OR = 0.13, 95% CrI 0.03–0.56) instead of TIVA. Moreover, when the largest study [17] was removed, we found the same significant association: reduction in mortality using halogenated (posterior mean of OR = 0.50, 95% CrI 0.28–0.96) and remote-volatile (posterior mean of OR = 0.16, 95% CrI 0.04–0.59) rather than TIVA.

The overall results were confirmed when we performed the network meta-analysis using trials with more than 80 patients.

Table 2 reports the posterior distribution of the probability to be the best and the worst for each anesthetic agent, showing a trend of both TIVA and remote-TIVA to be the worst in terms of short and long-term survival after cardiac surgery. S1 Fig shows the cumulative rank curves for each anesthetic agent: the remote-volatile treatment was the preferred one.

**Table 2:** Posterior distribution of the probability to be the best and the worst for each anesthetic agents, derived by Bayesian hierarchical model.

Anesthetic agents	Probability to be the best	Probability to be the second	Probability to be the third	Probability to be the worst	SUCRA index
Remote-Volatile	0,9646	0,02805	0,005187	0,002187	98,5
Volatile	0,01549	0,802	0,173	0,009503	60,8
Remote-TIVA	0,01918	0,1636	0,5309	0,2863	30,5
TIVA	0,0007	0,006303	0,2909	0,7021	10,2

TIVA: Total intravenous anesthesia

## Discussion

This Bayesian network meta-analysis demonstrates that in patients undergoing cardiac surgery the use of volatile agents (posterior mean of OR = 0.50, 95% CrI 0.28–0.91) and the combination of volatile agents with remote preconditioning (posterior mean of OR = 0.15, 95% CrI 0.04–0.55) are associated with a reduction in mortality at the longest follow-up available when compared to TIVA. This result is confirmed by repeating the analysis with low-risk-of-bias trials, removing the largest study, [17] and including only trials with more than 80 patients. On top of that, the concomitant use of volatile agents and remote preconditioning is associated with a reduction in mortality when compared with the concomitant use of TIVA and remote preconditioning.

Posterior distribution of the probability of each treatment to be the best one shows that the association volatile-remote is the best treatment to improve short- and long-term survival after cardiac surgery (Table 2, S1 Fig) suggesting an additive effect.

The effects of halogenated anesthetics in improving survival in cardiac surgery are not a new finding. It was first suggested in 2007 [70] and then confirmed several times thereafter,[71] the most recent confirmation being a meta-analysis of 38 randomized clinical trials showing that volatile agents were associated with a reduction in mortality when compared to TIVA (25/1994 (1.3%) in the volatile group vs. 43/1648 (2.6%) in the TIVA arm, OR = 0.51, 95% CI 0.33–0.81, p for effect = 0.004, number needed to treat 74, I-square = 0%).[7]

Remote preconditioning is a simple and non-invasive strategy that can protect myocardial tissue: a brief previous ischemic period can reduce tissue infarct size when a sustained and more intense ischemic insult is applied. In most trials included in our meta-analysis preconditioning was achieved through various cycles of mechanical limb ischemia with a pressure cuff or a tourniquet for some minutes.

A recent meta-analysis [3] suggested that remote ischemic preconditioning significantly reduces levels of postoperative myocardial injury biomarkers (standardized mean difference = -0.31, p for effect = 0.041; heterogeneity test: I-square = 83.5%) in adult cardiac surgery, but this cardioprotective effect seemed to be attenuated when combined with β-blockers or volatile anesthetics.

Another recent meta-analysis [4] of 19 randomized clinical trials involving 1,235 patients showed a statistically significant reduction in cardiac troponin I (cTnI) release at 6 hours post-operatively and in total cTnI release after cardiac surgery in patients who received remote ischemic preconditioning through limb ischemia versus control group (weighted mean difference -2.03ug/L, 95% CI -3.25 to -0.82ug/L, p for effect = 0.001; weighted mean difference -65.74ug/L*h, 95% CI -107.88 to -23.61ug/L*h, p for effect = 0.002, respectively), but with no differences in mortality.

Our findings suggest that volatile agents in the cardiac surgery setting are associated with a reduction in mortality and that the combination of volatile agents with remote ischemic preconditioning gives further protection.

Bayesian network meta-analysis is a research method that directly and indirectly compares different groups, with an established role in clinical research. We have included in our meta-analysis all randomized trials ever done in adult cardiac setting that compare volatile agents, TIVA and remote ischemic preconditioning. On the basis of these findings, we strongly encourage the use of volatile agents alone or associated with remote ischemic preconditioning in patients undergoing cardiac surgery.

Ischemic preconditioning was described for the first time by Murry et al [72] in 1986: they demonstrated that several short episodes of coronary occlusion and reperfusion were able to reduce myocardial infarct size when a sustained coronary occlusion occurred. The study of ischemia and reperfusion has been extended to human subjects and to other organs. The mechanism of preconditioning is still unclear but memory seems to be at the basis of this tissue protection: the heart remembers a previous insult and reacts to subsequent ischemia [73] by modifying gene expression and suppress pro-inflammatory response to ischemia-reperfusion damage. [74] Activation of various molecular pathways protects myocytes through an early preconditioning (for 1–6 hours), related to the mitochondrial KATP channel, δ -opioid and bradykinin receptors activation, and through a delayed phase (from 24–72 hours), related to the induction of nitric oxide synthase, superoxide dismutase and heat-shock proteins. Volatile agents mimic the early phase of ischemic preconditioning, activating mitochondrial KATP channels through a multi-pathway signaling. [8]

On the basis of our findings, general anesthesia in adult cardiac surgery should be maintained with volatile agents. In fact there is no evidence of any beneficial properties of propofol when compared to volatile agents on clinically relevant outcomes. Heusch et al recently discovered that protection by remote ischemic preconditioning is associated with the activation of signal transducer and activator of transcription 5 in left ventricular biopsy samples of patients undergoing coronary artery bypass grafting during isoflurane anesthesia. [75] In their more recent randomized trial, they hypothesized that propofol anesthesia interferes with signal transducer and activator of transcription 5 activation, but they failed to demonstrate it, probably because propofol interacts with cardioprotective signaling upstream of signal transducer and activator of transcription 5, that have yet to be identified. [76]

Application of mechanical ischemic preconditioning through a pressure cuff or tourniquet in patients under general anesthesia undergoing cardiac surgery is an economical and simple strategy that could change short and long term outcome, especially in high risk patients.

This meta-analysis presents some limitations: most of the included studies are small, single-center and not double blind. In some of them, authors do not clarify if patients that were taking drugs such as sulfonylurea, theophylline or allopurinol have been excluded; in studies in which patients were under these drugs, they do not specify the timing of preoperative withdrawal. These drugs, in fact, seem to interfere with preconditioning mechanism. Also data on intraoperative amount of opioids have not been clarified at all. Opioids reduce cardiovascular stress and could influence cardio-protective effects of volatile agents. Furthermore, the length of follow-up was not identical in the different RCTs included in the meta-analysis; nonetheless a metaregression suggested that the beneficial effect of volatile agents and remote ischemic preconditioning did not vary over time

Large, multicenter, randomized, double-blinded trials comparing these different strategies (volatile, remote and TIVA) in cardiac surgery are necessary to confirm our results. It is also necessary to establish which is the most protective volatile agent and in what manner remote ischemic preconditioning should be applied. These findings could be of great interest also in non-cardiac setting, especially in high risk patients, since major non cardiac surgery is often associated with myocardial damage, leading to prolonged hospital stay and increasing perioperative morbidity and mortality.

The limitations of Bayesian network meta-analyses are well known. [77] Traditional limitations of meta-analyses, due to variations in the treatment regimens or in the management of the trials also apply to Bayesian network meta-analyses. [78] Moreover, Bayesian network meta-analyses include direct and indirect comparisons among treatments. Indirect evidence is susceptible to confounding and therefore should be carefully interpreted. Also, even if the main effect of study precision was not associated with the risk of mortality, we cannot completely exclude the presence of publication bias.

## Conclusions

In patients undergoing cardiac surgery the use of volatile agents and the combination of volatile agents with remote preconditioning are associated with a reduction in mortality when compared to TIVA at the longest follow-up available. On the basis of our findings, general anesthesia in cardiac surgery should be performed with volatile agents because there is no evidence of any beneficial properties of total intravenous anesthesia alone compared with volatile agents. Furthermore, application of mechanical remote ischemic preconditioning is a simple strategy that could change short- and long-term outcome. It is necessary to confirm these results with large, multicenter, randomized, double-blinded trials comparing these different strategy (volatile, remote and TIVA) in cardiac and also in non-cardiac surgery, and to establish which volatile agent is more protective than the others and how to applied remote ischemic precondition.

## Supporting Information

S1 Checklist Checklist for transparent and complete reporting of systematic reviews and meta-analyses. (PDF)

S1 Fig Cumulative rank curve: cumulative probability to be the best, the second, the third and the worst. (PNG)

S2 Fig Forest plot of the risk of mortality: Halogenated vs total intravenous anesthesia. (PNG)

S3 Fig Forest plot of the risk of mortality: Volatile vs Remote-Volatile. (PNG)

S4 Fig Forest plot of the risk of mortality: TIVA vs Remote-TIVA. (PNG)

S5 Fig Forest plot of the risk of mortality: Halogenated vs Remote-TIVA. (PNG)

S1 File Search strategy. (DOCX)

S2 File WinBUGS code. (DOCX)

S1 Table Baseline characteristics and results of included trials. (DOCX)

S2 Table Methodological quality summary: review authors' judgments about each methodological quality item for each included study. (DOCX)

## References

[1] Domanski MJ, Mahaffey K, Hasselblad V, Brener SJ, Smith PK, Hillis G, et al Association of myocardial enzyme elevation and survival following coronary artery bypass graft surgery. JAMA. 2011;305: 585–591. 21304084 10.1001/jama.2011.99

[2] Heusch G, Schulz RJ. Remote preconditioning. Mol Cell Cardiol. 2002;34: 1279–1281. 10.1006/jmcc.2002.2093 12392984

[3] Zhou C, Liu Y, Yao Y, Zhou S, Fang N, Wang W, et al β-blockers and volatile anesthetics may attenuate cardioprotection by remote preconditioning in adult cardiac surgery: a meta-analysis of 15 randomized trials. J Cardiothorac Vasc Anesth. 2013;27: 305–311. 23276595 10.1053/j.jvca.2012.09.028

[4] Yang L, Wang G, Du Y, Ji B, Zheng Z. Remote Ischemic Preconditioning Reduces Cardiac Troponin I Release in Cardiac Surgery: A Meta-Analysis. J Cardiothorac Vasc Anesth. 2013;28: 682–689. 24103716 10.1053/j.jvca.2013.05.035

[5] Thielmann M, Kottenberg E, Kleinbongard P, Wendt D, Gedik N, Pasa S, et al Cardioprotective and prognostic effects of remote ischaemic preconditioning in patients undergoing coronary artery bypass surgery: a single-centre randomised, double-blind, controlled trial. Lancet. 2013;382: 597–604. 23953384 10.1016/S0140-6736(13)61450-6

[6] Landoni G, Augoustides JG, Guarracino F, Santini F, Ponschab M, Pasero D, et al Mortality reduction in cardiac anesthesia and intensive care: results of the first International Consensus Conference. Acta Anaesthesiol Scand. 2011;55: 259–266. 21288207 10.1111/j.1399-6576.2010.02381.x

[7] Landoni G, Greco T, Biondi-Zoccai G, Nigro Neto C, Febres D, Pintaudi M, et al Anaesthetic drugs and survival: a Bayesian network meta-analysis of randomized trials in cardiac surgery. Br J Anaesth. 2013;111: 886–896. 23852263 10.1093/bja/aet231

[8] Swyers T, Redford D, Larson DF. Volatile anesthetic-induced preconditioning. Perfusion. 2014;29: 10–15. 24002781 10.1177/0267659113503975

[9] Biondi-Zoccai GG, Agostoni P, Abbate A, Testa L, Burzotta F. A simple hint to improve Robinson and Dickersin's highly sensitive PubMed search strategy for controlled clinical trials. Int J Epidemiol. 2005;34: 224–225 15659479 10.1093/ije/dyh311

[10] Higgins JPT, Green S. The Cochrane Handbook for Systematic Reviews of Interventions. Version 5.0.2. Available: http://www.mrc-bsu.cam.ac.uk/cochrane/handbook502/

[11] Adamakis S, Raftery CL, Walsh RW, Gallagher PT. A Bayesian approach to comparing theoretic models to observational data: A case study from solar flare physics. astro-ph.SR. 2012; arXiv:1102.0242v3. Available: http://arxiv.org/abs/1102.0242

[12] Jansen JP, Fleurence R, Devine B, Itzler R, Barrett A, Hawkins N, et al Interpreting indirect treatment comparisons and network meta-analysis for health-care decision making: report of the ISPOR Task Force on Indirect Treatment Comparisons Good Research Practices: part 1. Value Health. 2011;14: 417–428. 21669366 10.1016/j.jval.2011.04.002

[13] Dias S, Welton NJ, Sutton AJ, Caldwell DM, Lu G, Ades AE. NICE DSU Technical Support Document 4: Inconsistency in networks of evidence based on randomised controlled trials. Available: http://www.nicedsu.org.uk 27466656

[14] Dias S, Welton, N, Sutton, AJ, Ades AE. NICE DSU Technical Support Document 2: A generalised linear modelling framework for pair-wise and network meta-analysis of Randomised Controlled Trials. Available: http://www.nicedsu.org.uk 27466657

[15] Amr YM, Yassin IM. Cardiac protection during on-pump coronary artery bypass grafting: ischemic versus isoflurane preconditioning. Semin Cardiothorac Vasc Anesth. 2010;14: 205–211. 20656748 10.1177/1089253210376839

[16] Kottenberg E, Thielmann M, Bergmann L, Heine T, Jakob H, Heusch G, et al Protection by remote ischemic preconditioning during coronary artery bypass graft surgery with isoflurane but not propofol—a clinical trial. Acta Anaesthesiol Scand. 2012;56: 30–38. 22103808 10.1111/j.1399-6576.2011.02585.x

[17] Hong DM, Mint JJ, Kim JH, Sohn IS, Lim TW, Lim YT, et al The effect of remote ischaemic preconditioning on myocardial injury in patients undergoing off-pump coronary artery bypass graft surgery. Anaesth Intensive Care. 2010;38: 924–929. 20865880 10.1177/0310057X1003800518

[18] Liberati A, Altman DG, Tetzlaff J, Mulrow C, Gøtzsche PC, Ioannidis JP, et al The PRISMA statement for reporting systematic reviews and meta-analyses of studies that evaluate healthcare interventions: explanation and elaboration. BMJ. 2009;339: b2700. 19622552 10.1136/bmj.b2700 PMC2714672

[19] Ballester M, Llorens J, Garcia-de-la-Asuncion J, Perez-Griera J, Tebar E, Martinez-Leon J, et al Myocardial oxidative stress protection by sevoflurane vs. propofol: a randomised controlled study in patients undergoing off-pump coronary artery bypass graft surgery. Eur J Anaesthesiol. 2011;28: 874–881. 21946824 10.1097/EJA.0b013e32834bea2a

[20] Bein B, Renner J, Caliebe D, Scholz J, Paris A, Fraund S, et al Sevoflurane but not propofol preserves myocardial function during minimally invasive direct coronary artery bypass surgery. Anesth Analg. 2005;100:610–616. 15728039 10.1213/01.ANE.0000145012.27484.A7

[21] Belhomme D, Peynet J, Louzy M, Launay JM, Kitakaze M, Menasché P. Evidence for preconditioning by isoflurane in coronary artery bypass graft surgery. Circulation. 1999;100: II340–344. 10567326 10.1161/01.cir.100.suppl_2.ii-340

[22] Bignami E, Landoni G, Gerli C, Testa V, Mizzi A, Fano G, et al Sevoflurane vs. propofol in patients with coronary disease undergoing mitral surgery: a randomised study. Acta Anaesthesiol Scand. 2012;56: 482–490. 22103571 10.1111/j.1399-6576.2011.02570.x

[23] Cavalca V, Colli S, Veglia F, Eligini S, Zingaro L, Squellerio I, et al Anesthetic propofol enhances plasma gamma-tocopherol levels in patients undergoing cardiac surgery. Anesthesiology. 2008;108: 988–997. 18497598 10.1097/ALN.0b013e318173efb4

[24] Choi YS, Shim JK, Kim JC, Kang KS, Seo YH, Ahn KR, et al Effect of remote ischemic preconditioning on renal dysfunction after complex valvular heart surgery: a randomized controlled trial. J Thorac Cardiovasc Surg. 2011;142: 148–154. 21272897 10.1016/j.jtcvs.2010.11.018

[25] Conzen PF, Fischer S, Detter C, Peter K. Sevoflurane provides greater protection of the myocardium than propofol in patients undergoing off-pump coronary artery bypass surgery. Anesthesiology. 2003;99: 826–833. 14508313 10.1097/00000542-200310000-00013

[26] Cromheecke S, Pepermans V, Hendrickx E, Lorsomradee S, Ten Broecke PW, Stockman BA, et al Cardioprotective properties of sevoflurane in patients undergoing aortic valve replacement with cardiopulmonary bypass. Anesth Analg. 2006;103: 289–296. 16861404 10.1213/01.ane.0000226097.22384.f4

[27] De Hert SG, Cromheecke S, ten Broecke PW, Mertens E, De Blier IG, Stockman BA, et al Effects of propofol, desflurane, and sevoflurane on recovery of myocardial function after coronary surgery in elderly high-risk patients. Anesthesiology. 2003;99: 314–323. 12883404 10.1097/00000542-200308000-00013

[28] De Hert SG, Van der Linden PJ, Cromheecke S, Meeus R, ten Broecke PW, De Blier IG, et al Choice of primary anesthetic regimen can influence intensive care unit length of stay after coronary surgery with cardiopulmonary bypass. Anesthesiology. 2004;101: 9–20. 15220766 10.1097/00000542-200407000-00005

[29] De Hert SG, Van der Linden PJ, Cromheecke S, Meeus R, Nelis A, Van Reeth V, et al Cardioprotective properties of sevoflurane in patients undergoing coronary surgery with cardiopulmonary bypass are related to the modalities of its administration. Anesthesiology. 2004;101: 299–310. 15277911 10.1097/00000542-200408000-00009

[30] De Hert S, Vlasselaers D, Barbé R, Ory JP, Dekegel D, Donnadonni R, et al A comparison of volatile and non volatile agents for cardioprotection during on-pump coronary surgery. Anaesthesia. 2009;64: 953–960. 19686479 10.1111/j.1365-2044.2009.06008.x

[31] Flier S, Post J, Concepcion AN, Kappen TH, Kalkman CJ, Buhre WF. Influence of propofol-opioid vs. isoflurane-opioid anaesthesia on postoperative troponin release in patients undergoing coronary artery bypass grafting. Br J Anaesth. 2010;105: 122–130. 20573633 10.1093/bja/aeq111

[32] Garcia C, Julier K, Bestmann L, Zollinger A, von Segesser LK, Pasch T, et al Preconditioning with sevoflurane decreases PECAM-1 expression and improves one-year cardiovascular outcome in coronary artery bypass graft surgery. Br J Anaesth. 2005;94: 159–165. 15556966 10.1093/bja/aei026

[33] Gozdzik W, Adamik B, Gomulkiewicz A, Gozdzik A, Dziegiel P. Upregulation of HMBG1 and TLR -4 cardiac right atrium mRNA expression during CABG surgery–the effects of sevoflurane conditioning and postconditioning. Abstracts presented at WCA 2012. Br J Anaesth. 2012;108: ii37–ii44 22493781

[34] Guarracino F, Landoni G, Tritapepe L, Pompei F, Leoni A, Aletti G, et al Myocardial damage prevented by volatile anesthetics: a multicenter randomized controlled study. J Cardiothorac Vasc Anesth. 2006;20: 477–483. 16884976 10.1053/j.jvca.2006.05.012

[35] Hellström J, Owall A, Sackey PV. Wake-up times following sedation with sevoflurane versus propofol after cardiac surgery. Scand Cardiovasc J. 2012;46: 262–268. 22420466 10.3109/14017431.2012.676209

[36] Helman JD, Leung JM, Bellows WH, Pineda N, Roach GW, Reeves JD 3rd, et al The risk of myocardial ischemia in patients receiving desflurane versus sufentanil anesthesia for coronary artery bypass graft surgery. The S.P.I. Research Group. Anesthesiology. 1992;77: 47–62. 1535185 10.1097/00000542-199207000-00008

[37] Hong DM, Jeon Y, Lee CS, Kim HJ, Lee JM, Bahk JH, et al Effects of remote ischemic preconditioning with postconditioning in patients undergoing off-pump coronary artery bypass surgery—randomized controlled trial. Circ J. 2012;76: 884–890. 22301846 10.1253/circj.cj-11-1068

[38] Hong DM, Lee EH, Kim HJ, Min JJ, Chin JH, Choi DK, et al Does remote ischaemic preconditioning with postconditioning improve clinical outcomes of patients undergoing cardiac surgery? Remote Ischaemic Preconditioning with Postconditioning Outcome Trial. Eur Heart J. 2014;35: 176–183. 24014392 10.1093/eurheartj/eht346

[39] Howie MB, Black HA, Romanelli VA, Zvara DA, Myerowitz PD, McSweeney TD. A comparison of isoflurane versus fentanyl as primary anesthetics for mitral valve surgery. Anesth Analg. 1996;83: 941–948. 8895267 10.1097/00000539-199611000-00009

[40] Huang Z, Zhong X, Irwin MG, Ji S, Wong GT, Liu Y, et al Synergy of isoflurane preconditioning and propofol postconditioning reduces myocardial reperfusion injury in patients. Clin Sci (Lond). 2011;121: 57–69. 21291422 10.1042/CS20100435

[41] Jovic M, Stancic A, Nenadic D, Cekic O, Nezic D, Milojevic P, et al Mitochondrial molecular basis of sevoflurane and propofol cardioprotection in patients undergoing aortic valve replacement with cardiopulmonary bypass. Cell Physiol Biochem. 2012;29: 131–142. 22415082 10.1159/000337594

[42] Kendall JB, Russell GN, Scawn ND, Akrofi M, Cowan CM, Fox MA. A prospective, randomised, single-blind pilot study to determine the effect of anaesthetic technique on troponin T release after off-pump coronary artery surgery. Anaesthesia. 2004;59: 545–549. 15144293 10.1111/j.1365-2044.2004.03713.x

[43] Kim JC, Shim JK, Lee S, Yoo YC, Yang SY, Kwak YL. Effect of combined remote ischemic preconditioning and postconditioning on pulmonary function in valvular heart surgery. Chest. 2012;142: 467–475. 22281799 10.1378/chest.11-2246

[44] Landoni G, Calabrò MG, Marchetti C, Bignami E, Scandroglio AM, Dedola E, et al Desflurane versus propofol in patients undergoing mitral valve surgery. J Cardiothorac Vasc Anesth. 2007;21: 672–677. 17905272 10.1053/j.jvca.2006.11.017

[45] Lee MC, Chen CH, Kuo MC, Kang PL, Lo A, Liu K. Isoflurane preconditioning-induced cardio-protection in patients undergoing coronary artery bypass grafting. Eur J Anaesthesiol. 2006;23: 841–847. 16507192 10.1017/S0265021506000354

[46] Leung JM, Goehner P, O'Kelly BF, Hollenberg M, Pineda N, Cason BA, et al Isoflurane anesthesia and myocardial ischemia: comparative risk versus sufentanil anesthesia in patients undergoing coronary artery bypass graft surgery. The SPI (Study of Perioperative Ischemia) Research Group. Anesthesiology. 1991;74: 838–847. 1826989

[47] Li L, Luo W, Huang L, Zhang W, Gao Y, Jiang H, et al Remote perconditioning reduces myocardial injury in adult valve replacement: a randomized controlled trial. J Surg Res. 2010;164: e21–e26. 20850778 10.1016/j.jss.2010.06.016

[48] Lomivorotov VV, Shmyrev VA, Nepomnyaschih VA, Ponomarev DN, Knyazkova LG, Lomivorotov VN, et al Remote ischaemic preconditioning does not protect the heart in patients undergoing coronary artery bypass grafting. Interact Cardiovasc Thorac Surg. 2012;15: 18–22. 22493101 10.1093/icvts/ivs118 PMC3380992

[49] Lucchinetti E, Hofer C, Bestmann L, Hersberger M, Feng J, Zhu M, et al Gene regulatory control of myocardial energy metabolism predicts postoperative cardiac function in patients undergoing off-pump coronary artery bypass graft surgery: inhalational versus intravenous anesthetics. Anesthesiology. 2007;106: 444–457. 17325502 10.1097/00000542-200703000-00008

[50] Meco M, Cirri S, Gallazzi C, Magnani G, Cosseta D. Desflurane preconditioning in coronary artery bypass graft surgery: a double-blinded, randomised and placebo-controlled study. Eur J Cardiothorac Surg. 2007;32: 319–325. 17574858 10.1016/j.ejcts.2007.05.005

[51] Meybohm P, Renner J, Broch O, Caliebe D, Albrecht M, Cremer J, et al Postoperative neurocognitive dysfunction in patients undergoing cardiac surgery after remote ischemic preconditioning: a double-blind randomized controlled pilot study. PLoS One. 2013;31: e64743. 10.1371/journal.pone.0064743 PMC3669352 23741380

[52] Musialowicz T, Niskanen M, Yppärilä-Wolters H, Pöyhönen M, Pitkänen O, Hynynen M. Auditory-evoked potentials in bispectral index-guided anaesthesia for cardiac surgery. Eur J Anaesthesiol. 2007;24: 571–579. 17462117 10.1017/S0265021507000403

[53] Rahman IA, Mascaro JG, Steeds RP, Frenneaux MP, Nightingale P, Gosling P, et al Remote ischemic preconditioning in human coronary artery bypass surgery: from promise to disappointment? Circulation. 2010;122: S53–S59. 20837926 10.1161/CIRCULATIONAHA.109.926667

[54] Royse CF, Andrews DT, Newman SN, Stygall J, Williams Z, Pang J, et al The influence of propofol or desflurane on postoperative cognitive dysfunction in patients undergoing coronary artery bypass surgery. Anaesthesia. 2011;66: 455–464. 21501129 10.1111/j.1365-2044.2011.06704.x

[55] Saxena P, Aggarwal S, Misso NL, Passage J, Newman MA, Thompson PJ, et al Remote ischaemic preconditioning down-regulates kinin receptor expression in neutrophils of patients undergoing heart surgery. Interact Cardiovasc Thorac Surg. 2013;17: 653–658. 23814135 10.1093/icvts/ivt279 PMC3781800

[56] Schoen J, Husemann L, Tiemeyer C, Lueloh A, Sedemund-Adib B, Berger KU, et al Cognitive function after sevoflurane- vs propofol-based anaesthesia for on-pump cardiac surgery: a randomized controlled trial. Br J Anaesth. 2011;106: 840–850. 21518736 10.1093/bja/aer091

[57] Soro M, Gallego L, Silva V, Ballester MT, Lloréns J, Alvariño A, et al Cardioprotective effect of sevoflurane and propofol during anaesthesia and the postoperative period in coronary bypass graft surgery: a double-blind randomised study. Eur J Anaesthesiol. 2012;29: 561–569. 22965457 10.1097/EJA.0b013e3283560aea

[58] Story DA, Poustie S, Liu G, McNicol PL. Changes in plasma creatinine concentration after cardiac anesthesia with isoflurane, propofol, or sevoflurane: a randomized clinical trial. Anesthesiology. 2001;95: 842–848. 11605922 10.1097/00000542-200110000-00010

[59] Tempe DK, Dutta D, Garg M, Minhas H, Tomar A, Virmani S. Myocardial protection with isoflurane during off-pump coronary artery bypass grafting: a randomized trial. J Cardiothorac Vasc Anesth. 2011;25: 59–65. 20580572 10.1053/j.jvca.2010.03.002

[60] Thielmann M, Kottenberg E, Boengler K, Raffelsieper C, Neuhaeuser M, Peters J, et al Remote ischemic preconditioning reduces myocardial injury after coronary artery bypass surgery with crystalloid cardioplegic arrest. Basic Res Cardiol. 2010;105: 657–664. 20495811 10.1007/s00395-010-0104-5

[61] Tritapepe L, Giorni C, Di Giovanni C, Pompei F, Cuscianna E, Pietropaoli P. Desflurane-sufentanil reduce troponin-I production after CABG. Eur J Anaesthesiol. 2003;20: A16.

[62] Tritapepe L, Landoni G, Guarracino F, Pompei F, Crivellari M, Maselli D, et al Cardiac protection by volatile anaesthetics: a multicentre randomized controlled study in patients undergoing coronary artery bypass grafting with cardiopulmonary bypass. Eur J Anaesthesiol. 2007;24: 323–331. 17156509 10.1017/S0265021506001931

[63] Wagner R, Piler P, Bedanova H, Adamek P, Grodecka L, Freiberger T. Myocardial injury is decreased by late remote ischaemic preconditioning and aggravated by tramadol in patients undergoing cardiac surgery: a randomised controlled trial. Interact Cardiovasc Thorac Surg. 2010;11: 758–762. 20847065 10.1510/icvts.2010.243600

[64] Williams JM, Young P, Pilcher J, Weatherall M, Miller JH, Beasley R, et al Remote ischaemic preconditioning does not alter perioperative cytokine production in high-risk cardiac surgery. Heart Asia. 2012: 97–101. 27326040 10.1136/heartasia-2012-010122 PMC4832611

[65] Wu Q, Gui P, Wu J, Ding D, Purusram G, Dong N, et al Effect of limb ischemic preconditioning on myocardial injury in patients undergoing mitral valve replacement surgery. A randomized controlled trial. Circ J. 2011;75: 1885–1889. 21697609 10.1253/circj.cj-10-1130

[66] Xie JJ, Liao XL, Chen WG, Huang DD, Chang FJ, Chen W, et al Remote ischaemic preconditioning reduces myocardial injury in patients undergoing heart valve surgery: randomised controlled trial. Heart. 2012;98: 384–388. 22107759 10.1136/heartjnl-2011-300860

[67] Yildirim V, Doganci S, Aydin A, Bolcal C, Demirkilic U, Cosar A. Cardioprotective effects of sevoflurane, isoflurane, and propofol in coronary surgery patients: a randomized controlled study. Heart Surg Forum. 2009;12: E1–E9. 19233758 10.1532/HSF98.20081137

[68] Young PJ, Dalley P, Garden A, Horrocks C, La Flamme A, Mahon B, et al A pilot study investigating the effects of remote ischemic preconditioning in high-risk cardiac surgery using a randomised controlled double-blind protocol. Basic Res Cardiol. 2012;107: 256. 22406977 10.1007/s00395-012-0256-6

[69] Zimmerman RF, Ezeanuna PU, Kane JC, Cleland CD, Kempananjappa TJ, Lucas FL, et al Ischemic preconditioning at a remote site prevents acute kidney injury in patients following cardiac surgery. Kidney Int. 2011;80: 861–867. 21677633 10.1038/ki.2011.156

[70] Landoni G, Biondi-Zoccai GG, Zangrillo A, Bignami E, D'Avolio S, Marchetti C, et al Desflurane and sevoflurane in cardiac surgery: a meta-analysis of randomized clinical trials. J Cardiothorac Vasc Anesth. 2007;21: 502–511. 17678775 10.1053/j.jvca.2007.02.013

[71] Bignami E, Greco T, Barile L, Silvetti S, Nicolotti D, Fochi O, et al The effect of isoflurane on survival and myocardial infarction: a meta-analysis of randomized controlled studies. J Cardiothorac Vasc Anesth. 2013;27: 50–58. 22819469 10.1053/j.jvca.2012.06.007

[72] Murry CE, Jennings RB, Reimer KA. Preconditioning with ischemia: a delay of lethal cell injury in ischemic myocardium. Circulation. 1986;74: 1124–1136. 3769170 10.1161/01.cir.74.5.1124

[73] Heush G, Schulz R. Remote precondition. J Mol Cell Cardiol. 2002;34: 1279–1281. 12392984 10.1006/jmcc.2002.2093

[74] Konstantinov IE, Arab S, Kharbanda RK, Li J, Cheung MM, Cherepanov V, et al The remote ischemic preconditioning stimulus modifies inflammatory gene expression in humans. Physiol Genomics. 2004;19: 143–150. 15304621 10.1152/physiolgenomics.00046.2004

[75] Heusch G, Musiolik J, Kottenberg E, Peters J, Jakob H, Thielmann M. STAT5 activation and cardioprotection by remote ischemic preconditioning in humans. Circ Res. 2012;110: 111–115. 22116817 10.1161/CIRCRESAHA.111.259556

[76] Kottenberg E, Musiolik J, Thielmann M, Jakob H, Peters J, Heusch G. Interference of propofol with signal transducer and activator of transcription 5 activation and cardioprotection by remote ischemic preconditioning during coronary artery bypass grafting. J Thorac Cardiovasc Surg. 2014;147: 376–382. 23465551 10.1016/j.jtcvs.2013.01.005

[77] Greco T, Biondi-Zoccai G, Saleh O, Pasin L, Cabrini L, Zangrillo A, et al The attractiveness of network meta-analysis: a comprehensive systematic and narrative review. Heart, Lung and Vessels. 2015. In press. PMC4476767 26157739

[78] Greco T, Zangrillo A, Biondi-ZoccaI G, Landoni G. Meta-analysis: pitfalls and hints. Heart, Lung and Vessels. 2013;5: 219–225. PMC3868184 24364016

