# Supplementary figures and images for: Additive Effect on Survival of Anaesthetic Cardiac Protection and Remote Ischemic Preconditioning in Cardiac Surgery: A Bayesian Network Meta-Analysis of Randomized Trials
Source: PLoS One. 2015 Jul 31;10(7):e0134264. doi: 10.1371/journal.pone.0134264 (PMC4521933; doi:10.1371/journal.pone.0134264)

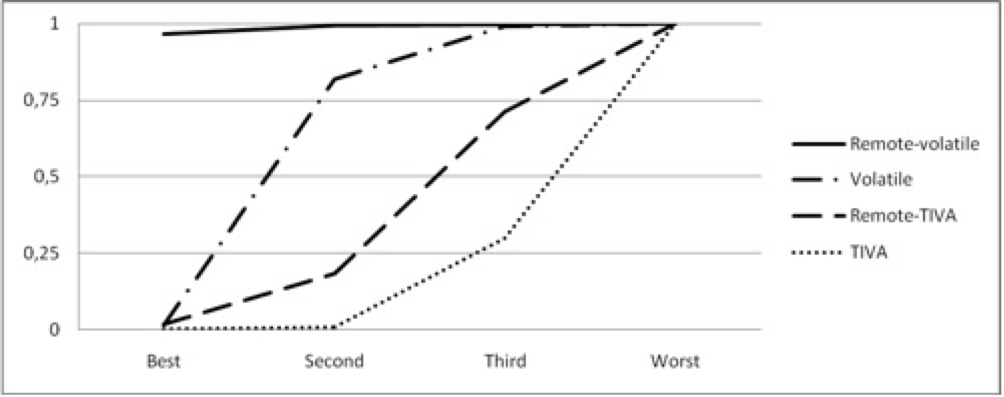

Supplement: S1 Fig — (PNG) [file pone.0134264.s002.png]

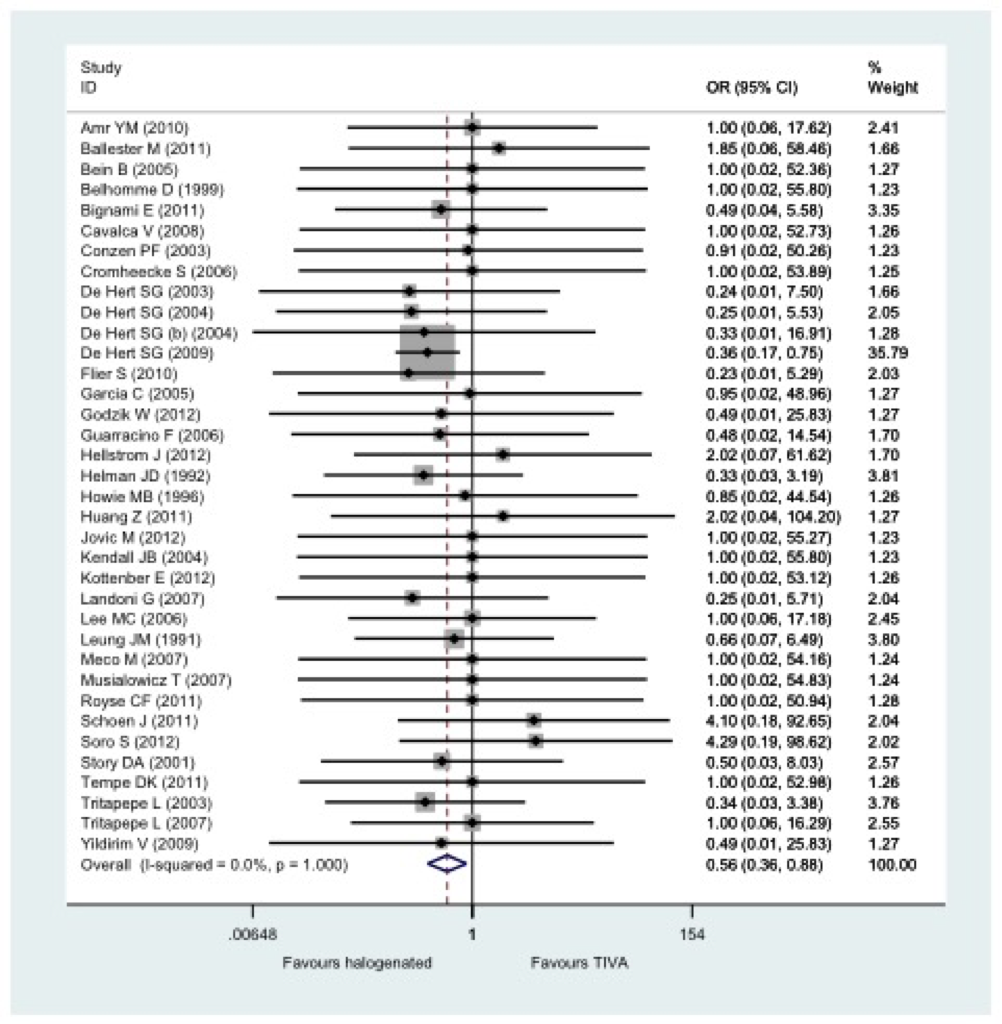

Supplement: S2 Fig — (PNG) [file pone.0134264.s003.png]

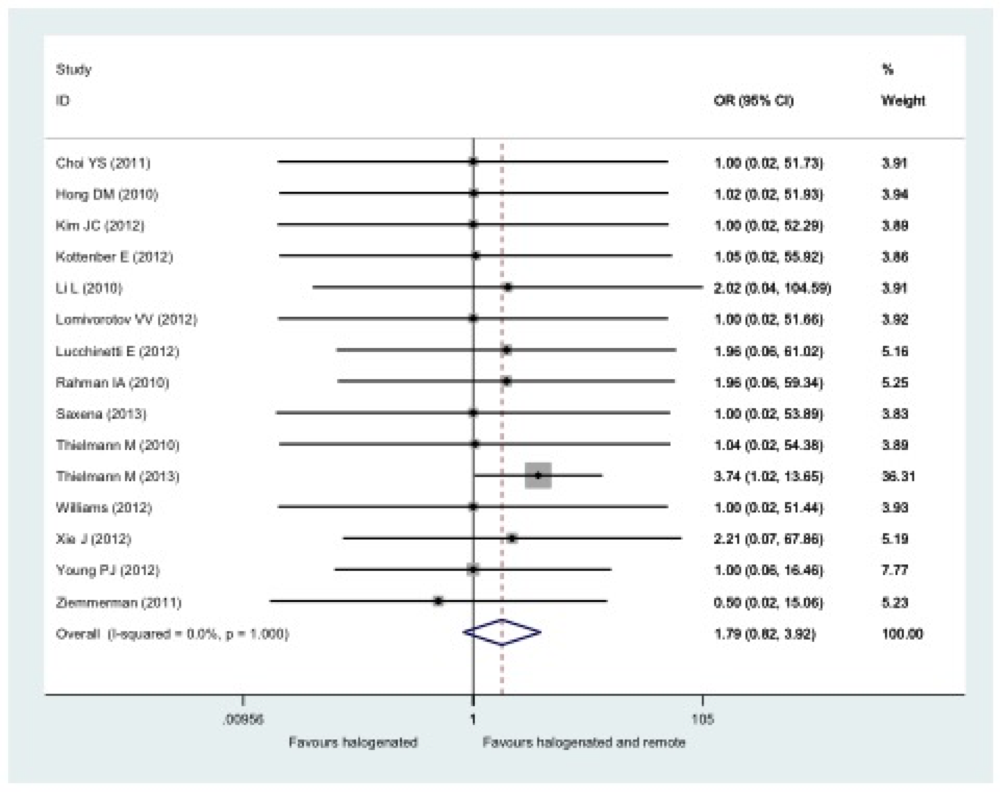

Supplement: S3 Fig — (PNG) [file pone.0134264.s004.png]

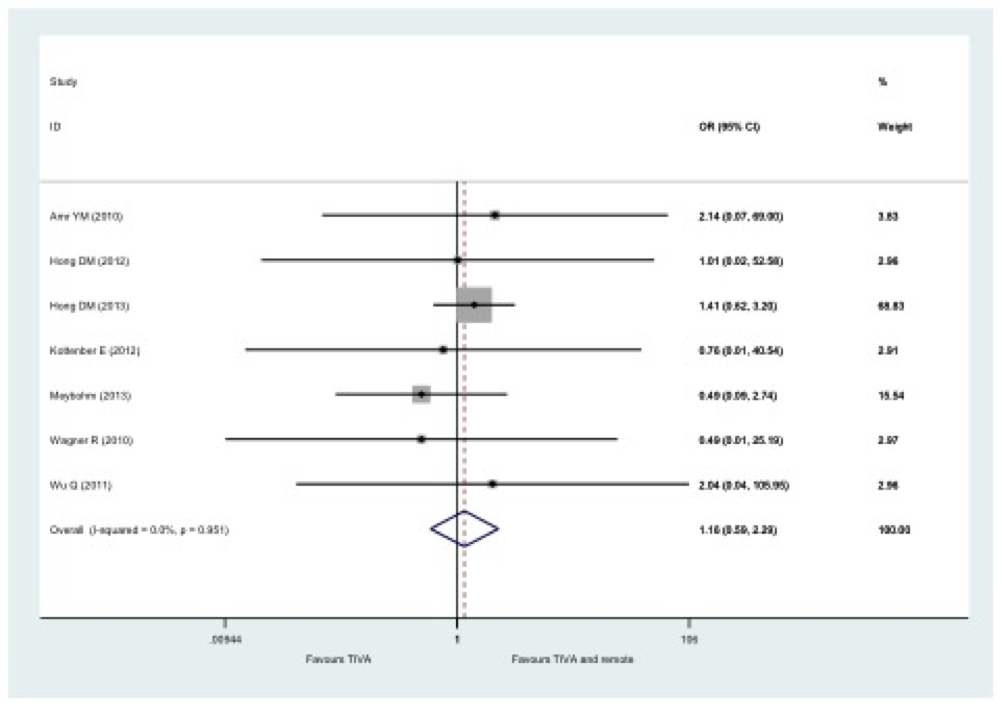

Supplement: S4 Fig — (PNG) [file pone.0134264.s005.png]

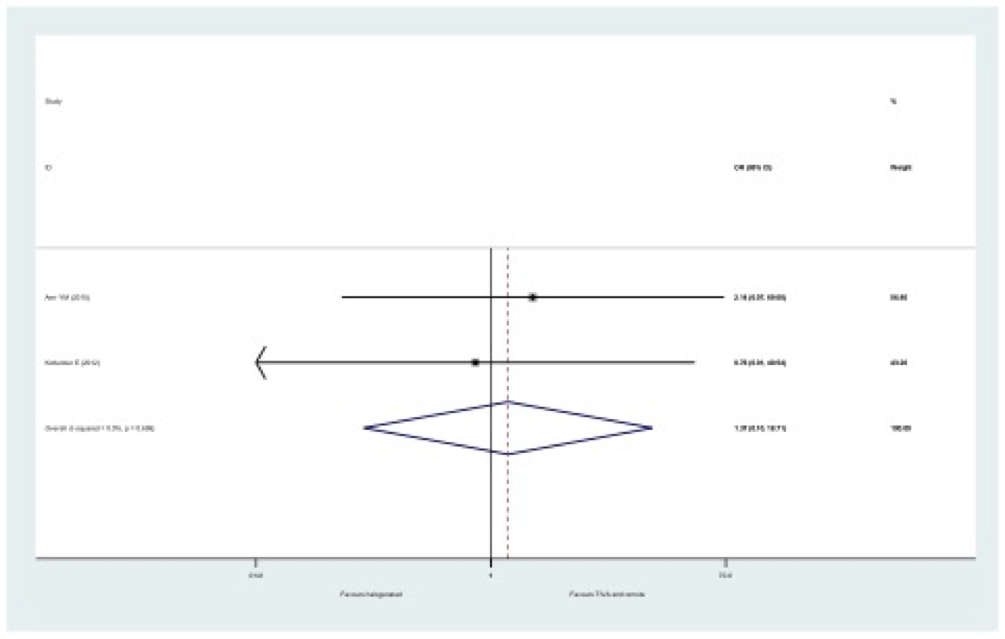

Supplement: S5 Fig — (PNG) [file pone.0134264.s006.png]
