# Supplementary material for: Additive Effect on Survival of Anaesthetic Cardiac Protection and Remote Ischemic Preconditioning in Cardiac Surgery: A Bayesian Network Meta-Analysis of Randomized Trials
Source: PLoS One. 2015 Jul 31;10(7):e0134264. doi: 10.1371/journal.pone.0134264 (PMC4521933; doi:10.1371/journal.pone.0134264)
Supplement: S1 File — (DOCX) [file pone.0134264.s007.docx]

**Additive effect on survival of anesthetic cardiac protection and remote ischemic preconditioning in cardiac surgery. A Bayesian network meta-analysis of randomized trials.**

*Zangrillo A, Musu M, Greco T, Di Prima AL, Matteazzi A, Testa V, Nardelli P, Febres D, Monaco F, Calabrò MG, Ma J, Finco G, Landoni G*

**Supporting Informations**

**Supplemental Material 1** Search strategy

**Supplemental Material 1** Search strategy

(heart OR cardiac OR myocard*OR coronary) AND (operatin*OR operation* OR surgery) AND (propofol OR isoflurane OR sevoflurane OR desflurane) AND (randomized controlled trial[pt] OR controlled clinical trial[pt] OR randomized controlled trials[mh] OR random allocation[mh] OR double-blind method[mh] OR single-blind method[mh] OR clinical trial[pt] OR clinical trials[mh] OR (clinical trial[tw] OR ((singl*[tw] OR doubl*[tw] OR trebl*[tw] OR tripl*[tw]) AND (mask*[tw] OR blind[tw])) OR (latin square[tw]) OR placebos[mh] OR placebo*[tw] OR random*[tw] OR research design[mh:noexp] OR comparative study[tw] OR follow-up studies[mh] OR prospective studies[mh] OR crossover studies[mh] OR control[tw] OR controls[tw] OR controlled[tw] OR prospectiv*[tw] OR volunteer*[tw]) NOT (animal[mh] NOT human[mh]) NOT (cavies OR rats OR pigs OR dogs)NOT (comment[pt] OR editorial[pt] OR meta-analysis[pt] OR practice-guideline[pt] OR review[pt]))
