# Supplementary material for: Additive Effect on Survival of Anaesthetic Cardiac Protection and Remote Ischemic Preconditioning in Cardiac Surgery: A Bayesian Network Meta-Analysis of Randomized Trials
Source: PLoS One. 2015 Jul 31;10(7):e0134264. doi: 10.1371/journal.pone.0134264 (PMC4521933; doi:10.1371/journal.pone.0134264)
Supplement: S2 File — (DOCX) [file pone.0134264.s008.docx]

**Additive effect on survival of anesthetic cardiac protection and remote ischemic preconditioning in cardiac surgery. A Bayesian network meta-analysis of randomized trials.**

*Zangrillo A, Musu M, Greco T, Di Prima AL, Matteazzi A, Testa V, Nardelli P, Febres D, Monaco F, Calabrò MG, Ma J, Finco G, Landoni G*

**Supporting Informations**

**Supplemental Material 2** WinBUGS code

**Supplemental Material 2** WinBUGS code

#MCT random effect model CONSISTENCY

model{

for(i in 1:ns){

w[i,1]<-0

delta[i,1]<-0

mu[i]~dnorm(0, 0.0001)

for(k in 1:na[i]){

r[i,k]~dbin(p[i,k], n[i,k]) #binomial likelihood

logit(p[i,k])<-mu[i]+delta[i,k] #model

}

for(k in 2:na[i]){

delta[i,k]~dnorm(md[i,k],taud[i,k]) #trial-specific LOR distribution

md[i,k]<-d[t[i,k]] - d[t[i,1]] + sw[i,k]

taud[i,k]<-tau *2*(k-1)/k #adjustament for multi-arm trial

w[i,k]<-(delta[i,k] - d[t[i,k]] + d[t[i,1]])

sw[i,k]<-sum(w[i,1:k-1])/(k-1)

}

}

d[1]<-0

for(k in 2:nt) {d[k]~dnorm(0,0.0001)}

tau~dgamma(0.001,0.001) #prior for 1/sigma2 (vantaggiosa nel caso di dati scarsi

sd<-pow(tau,-0.5) # more vague prior sd~dunif(0.5) tau<-pow(sd, -2)

for (k in 1:nt) {

rk[k]<-rank(d[], k)

best1[k]<-equals(rk[k],1)

best2[k]<-equals(rk[k],2)

best3[k]<-equals(rk[k],3)

best4[k]<-equals(rk[k],4)

}

}

#DATA

list(nt=4, ns=55,

na=c(3,2,2,2,2,2,2,2,2,2,2,2,2,2,2,2,2,2,2,2,2,2,2,2,2,2,2,4,2,2,2,2,2,2,2,2,2,2,2,2,2,2,2,2,2,2,2,2,2,2,2,2,2,2,2),

t= structure(

.Data=c(

1,2,3,NA,#Amr YM 2010

1,2,NA,NA,#Ballester M 2011

1,2,NA,NA,#Bein B 2005

1,2,NA,NA,#Belhomme D 1999

1,2,NA,NA,#Bignami E 2011

1,2,NA,NA,#Cavalca V 2008

2,4,NA,NA,#Choi YS 2011

1,2,NA,NA,#Conzen PF 2003

1,2,NA,NA,#Cromheecke S 2006

1,2,NA,NA,#De Hert SG 2003

1,2,NA,NA,#De Hert SG 2004

1,2,NA,NA,#De Hert SG 2009

1,2,NA,NA,#De Hert SG (b) 2004

1,2,NA,NA,#Flier S 2010

1,2,NA,NA,#Garcia C 2005

1,2,NA,NA,#Godzik W 2012

1,2,NA,NA,#Guarracino F 2006

1,2,NA,NA,#Hellstrom J 2012

1,2,NA,NA,#Helman JD 1992

2,4,NA,NA,#Hong DM 2010

1,3,NA,NA,#Hong DM 2012

1,3,NA,NA,#Hong DM 2013

1,2,NA,NA,#Howie MB 1996

1,2,NA,NA,#Huang Z 2011

1,2,NA,NA,#Jovic M 2004

1,2,NA,NA,#Kendall JB 2004

2,4,NA,NA,#Kim JC 2012

1,2,3,4,#Kottenber E 2012

1,2,NA,NA,#Landoni G 2007

1,2,NA,NA,#Lee MC 2006

1,2,NA,NA,#Leung JM 1991

2,4,NA,NA,#Li L 2010

2,4,NA,NA,#Lomivorotov VV 2012

2,4,NA,NA,#Lucchinetti E 2012

1,2,NA,NA,#Meco M 2007

1,3,NA,NA,#Meybohm 2013

1,2,NA,NA,#Musialowicz T 2007

2,4,NA,NA,#Rahman IA 2010

1,2,NA,NA,#Royse CF 2011

2,4,NA,NA,#Saxena 2013

1,2,NA,NA,#Schoen J 2011

1,2,NA,NA,#Soro S 2012

1,2,NA,NA,#Story DA 2001

1,2,NA,NA,#Tempe DK 2011

2,4,NA,NA,#Thielmann M 2010

2,4,NA,NA,#Thielmann M 2013

1,2,NA,NA,#Tritapepe L 2003

1,2,NA,NA,#Tritapepe L 2007

1,3,NA,NA,#Wagner R 2010

2,4,NA,NA,#Williams 2012

1,3,NA,NA,#Wu Q 2011

2,4,NA,NA,#Xie J 2012

1,2,NA,NA,#Yildirim V 2009

2,4,NA,NA,#young PJ 2012

2,4,NA,NA#Ziemmerman 2011

), .Dim=c(55,4)),

n= structure(

.Data=c(

15,15,15,NA,#Amr YM 2010

19,21,NA,NA,#Ballester M 2011

26,26,NA,NA,#Bein B 2005

10,10,NA,NA,#Belhomme D 1999

50,50,NA,NA,#Bignami E 2011

22,22,NA,NA,#Cavalca V 2008

38,38,NA,NA,#Choi YS 2011

11,12,NA,NA,#Conzen PF 2003

15,15,NA,NA,#Cromheecke S 2006

15,30,NA,NA,#De Hert SG 2003

160,160,NA,NA,#De Hert SG 2004

145,269,NA,NA,#De Hert SG 2009

50,150,NA,NA,#De Hert SG (b) 2004

49,51,NA,NA,#Flier S 2010

35,37,NA,NA,#Garcia C 2005

20,40,NA,NA,#Godzik W 2012

55,57,NA,NA,#Guarracino F 2006

50,50,NA,NA,#Hellstrom J 2012

100,100,NA,NA,#Helman JD 1992

66,67,NA,NA,#Hong DM 2010

35,35,NA,NA,#Hong DM 2012

663,665,NA,NA,#Hong DM 2013

23,27,NA,NA,#Howie MB 1996

60,30,NA,NA,#Huang Z 2011

11,11,NA,NA,#Jovic M 2004

10,10,NA,NA,#Kendall JB 2004

27,27,NA,NA,#Kim JC 2012

19,19,14,20,#Kottenber E 2012

61,59,NA,NA,#Landoni G 2007

20,20,NA,NA,#Lee MC 2006

124,62,NA,NA,#Leung JM 1991

27,54,NA,NA,#Li L 2010

40,40,NA,NA,#Lomivorotov VV 2012

28,27,NA,NA,#Lucchinetti E 2012

14,14,NA,NA,#Meco M 2007

90,90,NA,NA,#Meybohm 2013

12,12,NA,NA,#Musialowicz T 2007

82,80,NA,NA,#Rahman IA 2010

91,91,NA,NA,#Royse CF 2011

15,15,NA,NA,#Saxena 2013

64,64,NA,NA,#Schoen J 2011

37,36,NA,NA,#Soro S 2012

120,240,NA,NA,#Story DA 2001

20,20,NA,NA,#Tempe DK 2011

26,27,NA,NA,#Thielmann M 2010

167,162,NA,NA,#Thielmann M 2013

55,52,NA,NA,#Tritapepe L 2003

75,75,NA,NA,#Tritapepe L 2007

68,33,NA,NA,#Wagner R 2010

48,48,NA,NA,#Williams 2012

25,50,NA,NA,#Wu Q 2011

35,38,NA,NA,#Xie J 2012

20,40,NA,NA,#Yildirim V 2009

48,48,NA,NA,#young PJ 2012

60,60,NA,NA#Ziemmerman 2011

), .Dim=c(55,4)),

r= structure(

.Data=c(

1,1,0,NA,#Amr YM 2010

0,1,NA,NA,#Ballester M 2011

0,0,NA,NA,#Bein B 2005

0,0,NA,NA,#Belhomme D 1999

2,1,NA,NA,#Bignami E 2011

0,0,NA,NA,#Cavalca V 2008

0,0,NA,NA,#Choi YS 2011

0,0,NA,NA,#Conzen PF 2003

0,0,NA,NA,#Cromheecke S 2006

1,0,NA,NA,#De Hert SG 2003

2,0,NA,NA,#De Hert SG 2004

18,13,NA,NA,#De Hert SG 2009

0,0,NA,NA,#De Hert SG (b) 2004

2,0,NA,NA,#Flier S 2010

0,0,NA,NA,#Garcia C 2005

0,0,NA,NA,#Godzik W 2012

1,0,NA,NA,#Guarracino F 2006

0,1,NA,NA,#Hellstrom J 2012

3,1,NA,NA,#Helman JD 1992

0,0,NA,NA,#Hong DM 2010

0,0,NA,NA,#Hong DM 2012

14,10,NA,NA,#Hong DM 2013

0,0,NA,NA,#Howie MB 1996

0,0,NA,NA,#Huang Z 2011

0,0,NA,NA,#Jovic M 2004

0,0,NA,NA,#Kendall JB 2004

0,0,NA,NA,#Kim JC 2012

0,0,0,0,#Kottenber E 2012

2,0,NA,NA,#Landoni G 2007

1,1,NA,NA,#Lee MC 2006

3,1,NA,NA,#Leung JM 1991

0,0,NA,NA,#Li L 2010

0,0,NA,NA,#Lomivorotov VV 2012

1,0,NA,NA,#Lucchinetti E 2012

0,0,NA,NA,#Meco M 2007

2,4,NA,NA,#Meybohm 2013

0,0,NA,NA,#Musialowicz T 2007

1,0,NA,NA,#Rahman IA 2010

0,0,NA,NA,#Royse CF 2011

0,0,NA,NA,#Saxena 2013

0,2,NA,NA,#Schoen J 2011

0,2,NA,NA,#Soro S 2012

1,1,NA,NA,#Story DA 2001

0,0,NA,NA,#Tempe DK 2011

0,0,NA,NA,#Thielmann M 2010

11,3,NA,NA,#Thielmann M 2013

3,1,NA,NA,#Tritapepe L 2003

1,1,NA,NA,#Tritapepe L 2007

0,0,NA,NA,#Wagner R 2010

0,0,NA,NA,#Williams 2012

0,0,NA,NA,#Wu Q 2011

1,0,NA,NA,#Xie J 2012

0,0,NA,NA,#Yildirim V 2009

1,1,NA,NA,#young PJ 2012

0,1,NA,NA#Ziemmerman 2011

), .Dim=c(55,4))

)

#INITS MCT random effect model

#chain 1

list( d=c(NA, 0, 0, 0), tau=0.1, mu=c(0,0,0,0,0,0,0,0,0,0,0,0,0,0,0,0,0,0,0,0,0,0,0,0,0,0,0,0,0,0,0,0,0,0,0,0,0,0,0,0,0,0,0,0,0,0,0,0,0,0,0,0,0,0,0) )

#chain 2

list( d=c(NA, -1, 4, 2), tau=0.2, mu=c(2,-2,2,-2,2,-2,2,-2,2,-2,2,-2,2,-2,2,-2,2,-2,2,-2,2,-2,2,-2,2,-2,2,-2,2,-2,2,-2,2,-2,2,-2,2,-2,2,-2,2,-2,2,-2,2,-2,2,-2,2,-2,2,-2,2,-2,2))

#chain 3

list( d=c(NA, -2, 2, 2), tau=0.1, mu=c(-2,3,1,5,-4,0,-2,3,1,5,-4,0,-2,3,1,5,-4,0,-2,3,1,5,-4,0,-2,3,1,5,-4,0,-2,3,1,5,-4,0,-2,3,1,5,-4,0,-2,3,1,5,4,0,-2,3,1,5,-4,0,-2))
