# Supplementary material for: Additive Effect on Survival of Anaesthetic Cardiac Protection and Remote Ischemic Preconditioning in Cardiac Surgery: A Bayesian Network Meta-Analysis of Randomized Trials
Source: PLoS One. 2015 Jul 31;10(7):e0134264. doi: 10.1371/journal.pone.0134264 (PMC4521933; doi:10.1371/journal.pone.0134264)
Supplement: S1 Table — (DOCX) [file pone.0134264.s009.docx]

**Additive effect on survival of anesthetic cardiac protection and remote ischemic preconditioning in cardiac surgery. A Bayesian network meta-analysis of randomized trials.**

*Zangrillo A, Musu M, Greco T, Di Prima AL, Matteazzi A, Testa V, Nardelli P, Febres D, Monaco F, Calabrò MG, Ma J, Finco G, Landoni G*

**Supporting Informations**

**Supplemental Table 1** Baseline characteristics and results of included trials

**Supplemental Table 1** Baseline characteristics and results of included trials

| **Author** | **Year** | **Volatile agent** | **Comparison** | **Sample size** | **Dead and patients in volatile group** | **Dead and patients in TIVA group** | **Dead and patients in remote-volatile group** | **Dead and patients in remote-TIVA group** | **Longest follow-up** |
| --- | --- | --- | --- | --- | --- | --- | --- | --- | --- |
| Amr YM | 2010 | Isoflurane | Volatile **vs** TIVA **vs** remote-TIVA | 45 | 1/15 | 1/15 |  | 0/15 | 1 year |
| Ballester M | 2011 | Sevoflurane | Volatile **vs** TIVA | 40 | 1/21 | 0/19 |  |  | 1 year |
| Bein B | 2005 | Sevoflurane | Volatile **vs** TIVA | 52 | 0/26 | 0/26 |  |  | Hospital stay |
| Belhomme D | 1999 | Isoflurane | Volatile **vs** TIVA | 20 | 0/10 | 0/10 |  |  | 3 days |
| Bignami E | 2011 | Sevoflurane | Volatile **vs** TIVA | 100 | 1/50 | 1/50 |  |  | 1 year |
| Cavalca V | 2008 | Sevoflurane | Volatile **vs** TIVA | 44 | 0/22 | 0/22 |  |  | 24 hours |
| Choi YS | 2011 | Sevoflurane | Volatile **vs** remote-volatile | 76 | 0/38 |  | 0/38 |  | Hospital stay |
| Conzen PF | 2003 | Sevoflurane | Volatile **vs** TIVA | 23 | 0/12 | 0/11 |  |  | Hospital stay |
| Cromheecke S | 2006 | Sevoflurane | Volatile **vs** TIVA | 30 | 0/15 | 0/15 |  |  | Hospital stay |
| De Hert SG | 2003 | Sevoflurane, desflurane | Volatile **vs** TIVA | 45 | 0/30 | 1/15 |  |  | 36 hours |
| De Hert SG | 2004 | Sevoflurane, desflurane | Volatile **vs** TIVA | 320 | 0/160 | 2/160 |  |  | Hospital stay |
| De Hert SG (b) | 2004 | Sevoflurane | Volatile **vs** TIVA | 200 | 0/150 | 0/50 |  |  | 30 days |
| De Hert SG | 2009 | Sevoflurane, desflurane | Volatile **vs** TIVA | 414 | 13/269 | 18/145 |  |  | 1 year |
| Flier S | 2010 | Isoflurane | Volatile **vs** TIVA | 100 | 0/51 | 2/49 |  |  | 1 year |
| Garcia C | 2005 | Sevoflurane | Volatile **vs** TIVA | 72 | 0/37 | 0/35 |  |  | 1 year |
| Godzik W | 2012 | Sevoflurane | Volatile **vs** TIVA | 60 | 0/40 | 0/20 |  |  | 1 day |
| Guarracino F | 2006 | Desflurane | Volatile **vs** TIVA | 112 | 0/57 | 1/55 |  |  | 30 days |
| Hellstrom J | 2012 | Sevoflurane | Volatile **vs** TIVA | 100 | 1/50 | 0/50 |  |  | 30 days |
| Helman JD | 1992 | Desflurane | Volatile **vs** TIVA | 200 | 1/100 | 3/100 |  |  | 3 days |
| Hong DM | 2010 | Sevoflurane | Volatile **vs** remote-volatile | 133 | 0/66 |  | 0/67 |  | Hospital stay |
| Hong DM | 2012 | - | TIVA **vs** remote-TIVA | 70 |  | 0/35 |  | 0/35 | 30 days |
| Hong DM | 2013 | - | TIVA **vs** remote-TIVA | 1328 |  | 14/663 |  | 10/665 | Hospital stay |
| Howie MB | 1996 | Isoflurane | Volatile **vs** TIVA | 50 | 0/27 | 0/23 |  |  | 4 hours after surgical intensive care |
| Huang Z | 2011 | Isoflurane | Volatile **vs** TIVA | 90 | 0/30 | 0/60 |  |  | Hospital stay |
| Jovic M | 2012 | Sevoflurane | Volatile **vs** TIVA | 22 | 0/11 | 0/11 |  |  | Hospital stay |
| Kendall JB | 2004 | Isoflurane | Volatile **vs** TIVA | 20 | 0/10 | 0/10 |  |  | 48 hours |
| Kim JC | 2012 | NA | Volatile **vs** remote-volatile | 54 | 0/27 |  | 0/27 |  | Hospital stay |
| Kottenberg E | 2012 | Isoflurane | Volatile **vs** TIVA **vs** remote-volatile **vs** remote-TIVA | 72 | 0/19 | 0/19 | 0/20 | 0/14 | 72 hours |
| Landoni G | 2007 | Desflurane | Volatile **vs** TIVA | 120 | 0/59 | 2/61 |  |  | 30 days |
| Lee MC | 2006 | Isoflurane | Volatile **vs** TIVA | 40 | 1/20 | 1/20 |  |  | Hospital stay |
| Leung JM | 1991 | Isoflurane | Volatile **vs** TIVA | 186 | 1/62 | 3/124 |  |  | 3 days |
| Li L | 2010 | Isoflurane | Volatile **vs** remote-volatile | 81 | 0/27 |  | 0/54 |  | 30 days |
| Lomivorotov VV | 2012 | Isoflurane | Volatile **vs** remote-volatile | 80 | 0/40 |  | 0/40 |  | 48 hours |
| Lucchinetti E | 2012 | Isoflurane | Volatile **vs** remote-volatile | 55 | 1/28 |  | 0/27 |  | 6 months |
| Meco M | 2007 | Desflurane | Volatile **vs** TIVA | 28 | 0/14 | 0/14 |  |  | 72 hours |
| Meybohm | 2013 | - | TIVA **vs** remote-TIVA | 180 |  | 2/90 |  | 4/90 | 3 months |
| Musialowicz T | 2007 | Isoflurane | Volatile **vs** TIVA | 24 | 0/12 | 0/12 |  |  | End of surgery |
| Rahman IA | 2010 | Sevoflurane | Volatile **vs** remote-volatile | 162 | 1/82 |  | 0/80 |  | 30 days |
| Royse CF | 2011 | Desflurane | Volatile **vs** TIVA | 182 | 0/91 | 0/91 |  |  | 1 year |
| Saxena P | 2013 | NA | Volatile **vs** remote-volatile | 30 | 0/15 |  | 0/15 |  | Hospital stay |
| Schoen J | 2011 | Sevoflurane | Volatile **vs** TIVA | 128 | 2/64 | 0/64 |  |  | Hospital stay |
| Soro S | 2012 | Sevoflurane | Volatile **vs** TIVA | 73 | 2/36 | 0/37 |  |  | Hospital stay |
| Story DA | 2001 | Sevoflurane, isoflurane | Volatile **vs** TIVA | 360 | 1/240 | 1/120 |  |  | 3 days |
| Tempe DK | 2011 | Isoflurane | Volatile **vs** TIVA | 40 | 0/20 | 0/20 |  |  | 7 days |
| Thielmann M | 2010 | Isoflurane | Volatile **vs** remote-volatile | 53 | 0/26 |  | 0/27 |  | 30 days |
| Thielmann M | 2013 | Isoflurane | Volatile **vs** remote-volatile | 329 | 11/167 |  | 3/162 |  | 1 year |
| Tritapepe L | 2003 | Desflurane | Volatile **vs** TIVA | 107 | 1/52 | 3/55 |  |  | 30 days |
| Tritapepe L | 2007 | Desflurane | Volatile **vs** TIVA | 150 | 1/75 | 1/75 |  |  | ICU stay |
| Wagner R | 2010 | - | TIVA **vs** remote-TIVA | 101 |  | 0/68 |  | 0/33 | 24 hours |
| Williams JM | 2012 | Isoflurane | Volatile **vs** remote-volatile | 96 | 0/48 |  | 0/48 |  | 12 hours |
| Wu Q | 2011 | - | TIVA **vs** remote-TIVA | 75 |  | 0/25 |  | 0/50 | End of surgery |
| Xie J | 2012 | Sevoflurane | Volatile **vs** remote-volatile | 73 | 1/35 |  | 0/38 |  | 3 month |
| Yildirim V | 2009 | Sevoflurane, isoflurane | Volatile **vs** TIVA | 60 | 0/40 | 0/20 |  |  | 30 days |
| Young PJ | 2012 | Isoflurane | Volatile **vs** remote-volatile | 96 | 1/48 |  | 1/48 |  | 30 days |
| Ziemmerman | 2011 | Isoflurane | Volatile **vs** remote-volatile | 120 | 0/60 |  | 1/60 |  | Hospital stay |
