# Supplementary material for: Additive Effect on Survival of Anaesthetic Cardiac Protection and Remote Ischemic Preconditioning in Cardiac Surgery: A Bayesian Network Meta-Analysis of Randomized Trials
Source: PLoS One. 2015 Jul 31;10(7):e0134264. doi: 10.1371/journal.pone.0134264 (PMC4521933; doi:10.1371/journal.pone.0134264)
Supplement: S2 Table — (DOCX) [file pone.0134264.s010.docx]

**Additive effect on survival of anesthetic cardiac protection and remote ischemic preconditioning in cardiac surgery. A Bayesian network meta-analysis of randomized trials.**

*Zangrillo A, Musu M, Greco T, Di Prima AL, Matteazzi A, Testa V, Nardelli P, Febres D, Monaco F, Calabrò MG, Ma J, Finco G, Landoni G*

**Supporting Informations**

**Supplemental Table 2** Methodological quality summary: review authors' judgments about each methodological quality item for each included study.

**Supplemental Table 2** Methodological quality summary: review authors' judgments about each methodological quality item for each included study.

| **TRIAL** | **ENTRY** | **JUDGEMENT** | **DESCRIPTION** |
| --- | --- | --- | --- |
| Amr YM 2010 | Adequate sequence generation? | UNCLEAR | The manuscript doesn't report the sequence generation method. |
|  | Allocation concealment? | YES | Quote: "sealed envelopes indicated the group of assignment". |
|  | Blinding of participants and personnel? | UNCLEAR | There aren't sufficient information to permit judgement. |
|  | Blinding of outcome assessment? | UNCLEAR | There aren't sufficient information to permit judgement. |
|  | Complete outcome data addressed? | YES | No patients lost after randomization. |
|  | Free of selective reporting? | UNCLEAR | There aren't sufficient information to permit judgement. |
|  | Free of other bias? | YES | There is no evidence of other bias. |
|  | OVERALL RISK OF BIAS | LOW |  |
| Ballester M 2011 | Adequate sequence generation? | YES | Quote: “computer-generated random list” |
|  | Allocation concealment? | YES | Quote: "The randomisation allocation was concealed in an envelope until arrival in the operating room" |
|  | Blinding of participants and personnel? | YES | Single-blind. |
|  | Blinding of outcome assessment? | YES | Quote: "laboratory investigator were blinded to the anaesthetic agent used" |
|  | Complete outcome data addressed? | NO | Quote:"...38 of the 40 randomised patients successfully completed the study. None refused to participate in the study. Two patients were excluded, one because there was loss of the cold chain for the blood samples (propofol group) and the other was due to technical problems with haemodynamic monitoring that threatened standardisation of clinical management (sevoflurane group)." |
|  | Free of selective reporting? | UNCLEAR | There aren't sufficient information to permit judgement. |
|  | Free of other bias? | UNCLEAR | Quote: "First, it would have been interesting to check the differences between groups before and immediately after induction of anaesthesia, but coronary sinus samples were impossible to get at that time without another invasive intervention. Additionally, analysing oxidative stress markers in systemic blood even before induction might have helped to assess any potential relationship between their values and those in coronary sinus blood samples. Finally, it must be considered that our study was not powered to detect differences in cTnI levels, as the sample size was calculated on the basis of coronary sinus F2-isoprostanes concentrations" |
|  | OVERALL RISK OF BIAS | LOW |  |
| Bein B 2005 | Adequate sequence generation? | UNCLEAR | The manuscript doesn't report the sequence generation method. |
|  | Allocation concealment? | YES | Sealed envelopes. |
|  | Blinding of participants and personnel? | YES | Double-blind. |
|  | Blinding of outcome assessment? | YES | Quote:"Experienced investigator blinded as to the anesthetic used" |
|  | Complete outcome data addressed? | NO | Quote: "Two patients in the sevoflurane group were excluded because of an inability to obtain the intended TEE views" |
|  | Free of selective reporting? | UNCLEAR | There aren't sufficient information to permit judgement |
|  | Free of other bias? | YES | There is no evidence of other bias. |
|  | OVERALL RISK OF BIAS | LOW |  |
| Belhomme D 1999 | Adequate sequence generation? | UNCLEAR | The manuscript doesn't report the sequence generation method. |
|  | Allocation concealment? | UNCLEAR | No method reported for allocation concealment. |
|  | Blinding of participants and personnel? | UNCLEAR | There aren't sufficient information to permit judgement. |
|  | Blinding of outcome assessment? | UNCLEAR | There aren't sufficient information to permit judgement. |
|  | Complete outcome data addressed? | YES | No patients lost after randomization. |
|  | Free of selective reporting? | UNCLEAR | There aren't sufficient information to permit judgement. |
|  | Free of other bias? | YES | There is no evidence of other bias. |
|  | OVERALL RISK OF BIAS | MODERATE |  |
| Bignami E 2011 | Adequate sequence generation? | UNCLEAR | The manuscript doesn't report the sequence generation method. |
|  | Allocation concealment? | UNCLEAR | No method reported for allocation concealment. |
|  | Blinding of participants and personnel? | YES | Single-blind. |
|  | Blinding of outcome assessment? | UNCLEAR | There aren't sufficient information to permit judgement. |
|  | Complete outcome data addressed? | YES | Quote: "All 100 patients were analysed according to the intention to treat" |
|  | Free of selective reporting? | UNCLEAR | There aren't sufficient information to permit judgement. |
|  | Free of other bias? | YES | There is no evidence of other bias. |
|  | OVERALL RISK OF BIAS | LOW |  |
| Cavalca V 2008 | Adequate sequence generation? | YES | Quote: " random computer generated list" |
|  | Allocation concealment? | UNCLEAR | No method reported for allocation concealment. |
|  | Blinding of participants and personnel? | YES | Quote: "Anesthesiologists were unaware of the due treatment until the morning of surgery, after patient enrollment" |
|  | Blinding of outcome assessment? | UNCLEAR | There aren't sufficient information to permit judgement. |
|  | Complete outcome data addressed? | NO | One patiet excluded after randomization. |
|  | Free of selective reporting? | YES | Outcomes have been reported in the pre-specified way. |
|  | Free of other bias? | UNCLEAR | Quote: "Basic hemodynamic parameters could not be measured continuously in all patients because, according to the clinical practice of our institution, only selected patients had a Swan-Ganz catheter in place" |
|  | OVERALL RISK OF BIAS | MODERATE |  |
| Choi YS 2011 | Adequate sequence generation? | YES | Quote: "... patients were randomly assigned … by means of a computerized randomization table" |
|  | Allocation concealment? | UNCLEAR | No method reported for allocation concealment. |
|  | Blinding of participants and personnel? | YES | Quote: "Both cardiac surgeon and attending anesthesiologist were blinded to treatment allocation" |
|  | Blinding of outcome assessment? | UNCLEAR | There aren't sufficient information to permit judgement. |
|  | Complete outcome data addressed? | YES | No patients lost after randomization. |
|  | Free of selective reporting? | UNCLEAR | There aren't sufficient information to permit judgement. |
|  | Free of other bias? | NO | Quote: "The numbers of patients with diabetes and those undergoing reoperation for postoperative bleeding were higher in the control group, which may have confounded the results" |
|  | OVERALL RISK OF BIAS | MODERATE |  |
| Conzen PF 2003 | Adequate sequence generation? | UNCLEAR | The manuscript doesn't report the sequence generation method. |
|  | Allocation concealment? | UNCLEAR | No method reported for allocation concealment. |
|  | Blinding of participants and personnel? | UNCLEAR | There aren't sufficient information to permit judgement |
|  | Blinding of outcome assessment? | UNCLEAR | There aren't sufficient information to permit judgement |
|  | Complete outcome data addressed? | UNCLEAR | Quote: "Three additional patients met exclusion criteria after having been enrolled, i.e., were not included into the data analysis" |
|  | Free of selective reporting? | YES | Outcomes have been reported in the pre-specified way. |
|  | Free of other bias? | NO | Quote: "sevoflurane and propofol were used as a part of a multidrug anesthetic regimen" |
|  | OVERALL RISK OF BIAS | MODERATE |  |
| Cromheecke S 2006 | Adequate sequence generation? | YES | Quote: " A computer-generated random code determined which anesthetic protocol was identified by each treatment number" |
|  | Allocation concealment? | YES | Quote: "The participant randomization assignment was concealed in an envelope until the start of anesthesia" |
|  | Blinding of participants and personnel? | UNCLEAR | There aren't sufficient information to permit judgement |
|  | Blinding of outcome assessment? | YES | Quote: "Analysis of cardiac performance data was completed in a blinded fashion with the person completing the analysis having no knowledge of the anesthetic regimen used" |
|  | Complete outcome data addressed? | YES | No patients lost after randomization |
|  | Free of selective reporting? | UNCLEAR | There aren't sufficient information to permit judgement |
|  | Free of other bias? | NO | Quote: "Propofol and sevoflurane were used as a part of a multidrug anesthetic regimen. Opioids also have been shown to have a preconditioning effect. In the present study, anesthesia was based in part on a continuous infusion of remifentanil..." |
|  | OVERALL RISK OF BIAS | MODERATE |  |
| De Hert SG 2003 | Adequate sequence generation? | UNCLEAR | The manuscript doesn't report the sequence generation method. |
|  | Allocation concealment? | YES | Quote:"Patients were randomly (by opening of an envelope)" |
|  | Blinding of participants and personnel? | UNCLEAR | There aren't sufficient information to permit judgement. |
|  | Blinding of outcome assessment? | YES | Quote:"Analysis of cardiac performance data were completed in a blinded fashion with the person completing the analysis having no knowledge of the anesthetic regimen used in the patient" |
|  | Complete outcome data addressed? | NO | Quote: "One patient in the group A developed a myocardial infarction and died 24 h later. This patient was not included in the further analysis of hemodynamic and biochemical data. One patient in group B developed ventricular fibrillation after weaning from CPB, which was converted by defibrillation at 10 joules. This patient was also excluded from the study" |
|  | Free of selective reporting? | UNCLEAR | There aren't sufficient information to permit judgement. |
|  | Free of other bias? | YES | There is no evidence of other bias. |
|  | OVERALL RISK OF BIAS | MODERATE |  |
| De Hert SG 2004 | Adequate sequence generation? | YES | Quote: "A computer-generated random code determined which anesthetic protocol was identified by each treatment number" |
|  | Allocation concealment? | YES | Quote: "The participant randomization assignment was concealed in an envelope until the start of anesthesia" |
|  | Blinding of participants and personnel? | YES | Quote: "The surgeons, research assistants, and medical and nursing staff in the ICU and on the ward were blinded to the group assignments" |
|  | Blinding of outcome assessment? | YES | Quote: "The surgeons, research assistants, and medical and nursing staff in the ICU and on the ward were blinded to the group assignments" |
|  | Complete outcome data addressed? | YES | No patients lost after randomization. |
|  | Free of selective reporting? | UNCLEAR | There aren't sufficient information to permit judgement. |
|  | Free of other bias? | NO | Quote:"It cannot be excluded that some drugs with cardioprotective effect that were used as part of the routine surgical and anesthetic protocol may have shown some complex interaction with the anesthetic techniques used, and this could have influenced some of the results." |
|  | OVERALL RISK OF BIAS | LOW |  |
| De Hert SG (b) 2004 | Adequate sequence generation? | YES | Quote:"A computer-generated random code determined which anesthetic protocol was identified by each treatment number." |
|  | Allocation concealment? | YES | Quote: "The participant randomization assignment was concealed in an envelope" |
|  | Blinding of participants and personnel? | YES | Quote: "The surgeons, research assistants, and medical and nursing staff in the intensive care unit and the ward were blinded to the group assignments" |
|  | Blinding of outcome assessment? | UNCLEAR | There aren't sufficient information to permit judgement, |
|  | Complete outcome data addressed? | YES | No patients lost after randomization. |
|  | Free of selective reporting? | UNCLEAR | There aren't sufficient information to permit judgement, |
|  | Free of other bias? | YES | There is no evidence of other bias. |
|  | OVERALL RISK OF BIAS | LOW |  |
| De Hert SG 2009 | Adequate sequence generation? | YES | Quote: "A computerised block randomisation" |
|  | Allocation concealment? | UNCLEAR | No method reported for allocation concealment |
|  | Blinding of participants and personnel? | UNCLEAR | There aren't sufficient information to permit judgement |
|  | Blinding of outcome assessment? | UNCLEAR | There aren't sufficient information to permit judgement |
|  | Complete outcome data addressed? | YES | Quote: "Data were analysed on an intention-to-treat basis" |
|  | Free of selective reporting? | UNCLEAR | There aren't sufficient information to permit judgement |
|  | Free of other bias? | YES | There is no evidence of other bias. |
|  | OVERALL RISK OF BIAS | LOW |  |
| Flier S 2010 | Adequate sequence generation? | YES | Quote: "an envelope was randomly chosen and was taken to the operating theatre by one of the investigators" |
|  | Allocation concealment? | YES | Quote: "Randomization was performed using a sealed envelope" |
|  | Blinding of participants and personnel? | YES | Quote: "Patients, data collectors, and data analysers were blinded for the type of anaesthesia used" |
|  | Blinding of outcome assessment? | YES | Quote: "Patients, data collectors, and data analysers were blinded for the type of anaesthesia used" |
|  | Complete outcome data addressed? | NO | Quote: "In 13 patients, the study was discontinued and three patients were excluded from analysis" |
|  | Free of selective reporting? | YES | Outcomes have been reported in the pre-specified way. |
|  | Free of other bias? | NO | Quote: "Diabetic patients treated with sulphonylurea drugs were not excluded from the present study. Non-selective sulphonylurea derivatives, however, can block cardioprotective effects of anaesthetics by blocking the KATP channel and could have abolished the cardioprotective effect of isoflurane in the present study" |
|  | OVERALL RISK OF BIAS | LOW |  |
| Garcia C 2005 | Adequate sequence generation? | UNCLEAR | The manuscript doesn't report the sequence generation method. |
|  | Allocation concealment? | YES | Quote: "Patients were allocated randomly … using a sealed envelope technique." |
|  | Blinding of participants and personnel? | YES | Quote: "The surgeons, anaesthetists and perfusionists were blinded to the treatment" |
|  | Blinding of outcome assessment? | UNCLEAR | There aren't sufficient information to permit judgement. |
|  | Complete outcome data addressed? | YES | No patient lost after randomization. |
|  | Free of selective reporting? | UNCLEAR | There aren't sufficient information to permit judgement. |
|  | Free of other bias? | YES | There is no evidence of other bias. |
|  | OVERALL RISK OF BIAS | LOW |  |
| Gozdzik W 2012 (abstract) | Adequate sequence generation? | UNCLEAR | The manuscript doesn't report the sequence generation method. |
|  | Allocation concealment? | UNCLEAR | No method reported for allocation concealment. |
|  | Blinding of participants and personnel? | UNCLEAR | There aren't sufficient information to permit judgement. |
|  | Blinding of outcome assessment? | UNCLEAR | There aren't sufficient information to permit judgement. |
|  | Complete outcome data addressed? | UNCLEAR | There aren't sufficient information to permit judgement. |
|  | Free of selective reporting? | UNCLEAR | There aren't sufficient information to permit judgement. |
|  | Free of other bias? | UNCLEAR | There aren't sufficient information to permit judgement. |
|  | OVERALL RISK OF BIAS | MODERATE |  |
| Guarracino F 2006 | Adequate sequence generation? | YES | Quote:"The details of the randomization, created by a computer-generated list" |
|  | Allocation concealment? | YES | Quote:"… were contained in a set of sealed, opaque envelopes that were opened at the beginning of the anesthetic." |
|  | Blinding of participants and personnel? | YES | Quote: "Medical treatment and decision making in the ICU and in the ward were performed by physicians who were blinded to the anesthetic regimen used" |
|  | Blinding of outcome assessment? | YES | Quote: "Data were collected by trained observers who did not participate in patient care and who were blinded to the anesthetic regimen used" |
|  | Complete outcome data addressed? | YES | Quote: "All 112 patients were analyzed according to the intention-to-treat principle" |
|  | Free of selective reporting? | UNCLEAR | There aren't sufficient information to permit judgement |
|  | Free of other bias? | YES | There is no evidence of other bias. |
|  | OVERALL RISK OF BIAS | LOW |  |
| Hellstrom J 2012 | Adequate sequence generation? | UNCLEAR | The manuscript doesn't report the sequence generation method. |
|  | Allocation concealment? | YES | Quote: "… randomized sealed envelope" |
|  | Blinding of participants and personnel? | NO | Quote: "The study was not double-blinded, due to practical reasons with the new sevoflurane delivery device" |
|  | Blinding of outcome assessment? | UNCLEAR | There aren't sufficient information to permit judgement. |
|  | Complete outcome data addressed? | UNCLEAR | Quote: "Failed to receive intervention (n=1)" |
|  | Free of selective reporting? | UNCLEAR | There aren't sufficient information to permit judgement even if this randomized controlled study was registered as a clinical trial (Clinicaltrials.gov identifi er NCT- 00484575) and took place in the Cardiothoracic ICU at the Karolinska University Hospital, Solna. |
|  | Free of other bias? | NO | Quote: "… our propofol starting dose might have been higher than in some other units, potentially affecting wake-up times" |
|  | OVERALL RISK OF BIAS | MODERATE |  |
| Helman JD 1992 | Adequate sequence generation? | UNCLEAR | The manuscript doesn't report the sequence generation method. |
|  | Allocation concealment? | UNCLEAR | No method reported for allocation concealment. |
|  | Blinding of participants and personnel? | UNCLEAR | There aren't sufficient information to permit judgement. |
|  | Blinding of outcome assessment? | UNCLEAR | There aren't sufficient information to permit judgement. |
|  | Complete outcome data addressed? | YES | No patients lost after randomization. |
|  | Free of selective reporting? | UNCLEAR | There aren't sufficient information to permit judgement. |
|  | Free of other bias? | NO | No uniform distribution between males and females. |
|  | OVERALL RISK OF BIAS | MODERATE |  |
| Hong DM 2010 | Adequate sequence generation? | YES | Quote: "Patients were randomly allocated to the RIPC or control group by a computer-generated random code. |
|  | Allocation concealment? | UNCLEAR | No method reported for allocation concealment. |
|  | Blinding of participants and personnel? | YES | Quote: "The surgeons and anaesthesiologists, including the researchers, were blinded to the group assignments" |
|  | Blinding of outcome assessment? | YES | Quote: "The surgeons and anaesthesiologists, including the researchers, were blinded to the group assignments" |
|  | Complete outcome data addressed? | NO | Three patients lost after randomization. |
|  | Free of selective reporting? | UNCLEAR | There aren't sufficient information to permit judgement. |
|  | Free of other bias? | NO | Quote: "In the RIPC group, troponin levels were consistently lower than in the control group and the total amount of troponin release (AUC) was reduced by 26%" |
|  | OVERALL RISK OF BIAS | MODERATE |  |
| Hong DM 2012 | Adequate sequence generation? | YES | Quote: "Patients were randomly assigned to an RIPC+RIPostC group or to a control group using a computer- generated random list" |
|  | Allocation concealment? | UNCLEAR | Quote:" … and numbered envelopes." |
|  | Blinding of participants and personnel? | YES | Quote: "Surgeons and anesthesiologists were not told of patient assignments" |
|  | Blinding of outcome assessment? | UNCLEAR | There aren't sufficient information to permit judgement. |
|  | Complete outcome data addressed? | YES | No patient lost after randomization. |
|  | Free of selective reporting? | UNCLEAR | There aren't sufficient information to permit judgement. |
|  | Free of other bias? | NO | A lot of topics present in paragraph "study limitation", i.e. quote:"In the present study, we included patients with several factors, such as, old age, diabetes mellitus, hypertension, dyslipidemia, and medications, which might have modified the preconditioning effect." |
|  | OVERALL RISK OF BIAS | MODERATE |  |
| Hong DM 2014 | Adequate sequence generation? | YES | Quote: "Eligible patients were randomly allocated to either the RIPC with RIPostC group or the control group using a computer-generated list." |
|  | Allocation concealment? | YES | Quote: "The randomization list was generated by an independent statistician and was stored in concealed envelopes. |
|  | Blinding of participants and personnel? | YES | Quote: "The group assignment was performed in the morning of the surgery and was blinded to all patients, medical personnel, and investigators" |
|  | Blinding of outcome assessment? | UNCLEAR | There aren't sufficient information to permit judgement. |
|  | Complete outcome data addressed? | YES | Quote: "Data for all enrolled patients were included in the analysis of the primary and secondary endpoints according to the intention-to-treat principle" |
|  | Free of selective reporting? | UNCLEAR | There aren't sufficient information to permit judgement |
|  | Free of other bias? | YES | There is no evidence of other bias. |
|  | OVERALL RISK OF BIAS | LOW |  |
| Howie MB 1996 | Adequate sequence generation? | UNCLEAR | The manuscript doesn't report the sequence generation method. |
|  | Allocation concealment? | UNCLEAR | No method reported for allocation concealment |
|  | Blinding of participants and personnel? | UNCLEAR | There aren't sufficient information to permit judgement |
|  | Blinding of outcome assessment? | UNCLEAR | There aren't sufficient information to permit judgement |
|  | Complete outcome data addressed? | YES | No patients lost after randomization. |
|  | Free of selective reporting? | UNCLEAR | There aren't sufficient information to permit judgement |
|  | Free of other bias? | YES | There is no evidence of other bias. |
|  | OVERALL RISK OF BIAS | MODERATE |  |
| Huang Z 2011 | Adequate sequence generation? | YES | Quote: "were assigned according to a computer-generated random code" |
|  | Allocation concealment? | NO | Quote: "Subjects were assigned treatment numbers in ascending chronological order of admission in the study" |
|  | Blinding of participants and personnel? | YES | Quote: "The surgeons, research assistants and medical and nursing staff in the operation room were blinded to the group assignments" |
|  | Blinding of outcome assessment? | UNCLEAR | There aren't sufficient information to permit judgement |
|  | Complete outcome data addressed? | NO | One patient lost after randomization. Quote: "This patient suffered from acute perioperative myocardial infarction and died of severe right heart failure on day 2 after surgery. This patient’s data were excluded from statistical analysis." |
|  | Free of selective reporting? | UNCLEAR | There aren't sufficient information to permit judgement |
|  | Free of other bias? | YES | There is no evidence of other bias. |
|  | OVERALL RISK OF BIAS | MODERATE |  |
| Jovic M 2004 | Adequate sequence generation? | UNCLEAR | The manuscript doesn't report the sequence generation method. |
|  | Allocation concealment? | UNCLEAR | No method reported for allocation concealment. |
|  | Blinding of participants and personnel? | UNCLEAR | There aren't sufficient information to permit judgement. |
|  | Blinding of outcome assessment? | UNCLEAR | There aren't sufficient information to permit judgement. |
|  | Complete outcome data addressed? | YES | No patient lost after randomization. |
|  | Free of selective reporting? | UNCLEAR | There aren't sufficient information to permit judgement. |
|  | Free of other bias? | UNCLEAR | There aren't sufficient information to permit judgement. |
|  | OVERALL RISK OF BIAS | MODERATE |  |
| Kendall JB 2004 | Adequate sequence generation? | UNCLEAR | Quote: "Patients were randomly allocated to one of three groups using a shuffled, sealed envelope technique" |
|  | Allocation concealment? | YES | Quote: "… sealed envelope technique" |
|  | Blinding of participants and personnel? | NO | Single-blind pilot study. |
|  | Blinding of outcome assessment? | NO | There aren't sufficient information to permit judgement. |
|  | Complete outcome data addressed? | YES | No patient lost after randomization. |
|  | Free of selective reporting? | UNCLEAR | There aren't sufficient information to permit judgement. |
|  | Free of other bias? | YES | There is no evidence of other bias. |
|  | OVERALL RISK OF BIAS | MODERATE |  |
| Kim JC 2012 | Adequate sequence generation? | YES | Quote: "Patients were randomly assigned to ... according to a computerized randomization table" |
|  | Allocation concealment? | UNCLEAR | No method reported for allocation concealment. |
|  | Blinding of participants and personnel? | YES | Quote: "both attending anesthesiologists and cardiac surgeons were blinded to intervention allocation" |
|  | Blinding of outcome assessment? | UNCLEAR | There aren't sufficient information to permit judgement. |
|  | Complete outcome data addressed? | YES | No patient lost after randomization. |
|  | Free of selective reporting? | UNCLEAR | There aren't sufficient information to permit judgement even if this intervention protocol is registered at ClinicalTrials.gov (No: NCT01427621). |
|  | Free of other bias? | YES | There is no evidence of other bias. |
|  | OVERALL RISK OF BIAS | LOW |  |
| Kottenber E 2012 | Adequate sequence generation? | YES | Quote: "Before the trial, computer-generated randomization schedules were generated" |
|  | Allocation concealment? | YES | Quote: " and placed in sequentially numbered sealed envelopes" |
|  | Blinding of participants and personnel? | YES | Quote: " Laboratory personal measuring and providing troponin concentrations, patients, surgeons, echocardiographers, and critical care teams were blind as to the treatments and type of anesthesia assigned for the duration of the study" |
|  | Blinding of outcome assessment? | NO | Quote: "The anesthetist who applied the protocol but had no part in data sampling or analysis was not blind." |
|  | Complete outcome data addressed? | YES | No patient lost after randomization. |
|  | Free of selective reporting? | UNCLEAR | There aren't sufficient information to permit judgement |
|  | Free of other bias? | YES | There is no evidence of other bias. |
|  | OVERALL RISK OF BIAS | LOW |  |
| Landoni G 2007 | Adequate sequence generation? | YES | Quote: " The details of the randomization, created by a computer-generated list" |
|  | Allocation concealment? | YES | Quote: "… sealed, opaque envelopes that were opened at the beginning of the anesthetic" |
|  | Blinding of participants and personnel? | YES | Quote: "All study personnel, including those involved in cTnI measurement, were blinded to treatment assignment for the duration of the study" |
|  | Blinding of outcome assessment? | UNCLEAR | There aren't sufficient information to permit judgement. |
|  | Complete outcome data addressed? | YES | Quote: "All 120 patients were analyzed according to the intention-to-treat principle" |
|  | Free of selective reporting? | UNCLEAR | There aren't sufficient information to permit judgement |
|  | Free of other bias? | YES | There is no evidence of other bias. |
|  | OVERALL RISK OF BIAS | LOW |  |
| Lee MC 2006 | Adequate sequence generation? | UNCLEAR | The manuscript doesn't report the sequence generation method. |
|  | Allocation concealment? | UNCLEAR | There aren't sufficient information to permit judgement. |
|  | Blinding of participants and personnel? | UNCLEAR | There aren't sufficient information to permit judgement. |
|  | Blinding of outcome assessment? | UNCLEAR | There aren't sufficient information to permit judgement |
|  | Complete outcome data addressed? | YES | No patients lost after randomization. |
|  | Free of selective reporting? | UNCLEAR | There aren't sufficient information to permit judgement |
|  | Free of other bias? | NO | Quote: "Due to coronary artery disease and myocardial infarction occur with increased frequencies among diabetic patients, preconditioning of the diabetic myocardium may differ considerably from preconditioning of non-diabetic myocardium" |
|  | OVERALL RISK OF BIAS | MODERATE |  |
| Leung JM 1991 | Adequate sequence generation? | UNCLEAR | The manuscript doesn't report the sequence generation method. |
|  | Allocation concealment? | UNCLEAR | There aren't sufficient information to permit judgement. |
|  | Blinding of participants and personnel? | UNCLEAR | There aren't sufficient information to permit judgement. |
|  | Blinding of outcome assessment? | UNCLEAR | There aren't sufficient information to permit judgement. |
|  | Complete outcome data addressed? | NO | Data were not analyzed according to the intention-to-treat principle. |
|  | Free of selective reporting? | YES | Outcomes have been reported in the pre-specified way. |
|  | Free of other bias? | NO | No uniform distribution between males and females (185 men and 1 woman) |
|  | OVERALL RISK OF BIAS | MODERATE |  |
| Li L 2010 | Adequate sequence generation? | YES | Quote: "Eighty-one qualified patients were randomized to three groups by a computer-generated number table" |
|  | Allocation concealment? | UNCLEAR | No method reported for allocation concealment. |
|  | Blinding of participants and personnel? | YES | Quote: "Staff involved in the clinical care, and members collecting and analyzing data were blind to group allocation" |
|  | Blinding of outcome assessment? | YES | Quote: "Staff involved in the clinical care, and members collecting and analyzing data were blind to group allocation" |
|  | Complete outcome data addressed? | YES | No patient lost after randomization. |
|  | Free of selective reporting? | UNCLEAR | There aren't sufficient information to permit judgement |
|  | Free of other bias? | YES | There is no evidence of other bias. |
|  | OVERALL RISK OF BIAS | LOW |  |
| Lomivorotov VV 2012 | Adequate sequence generation? | UNCLEAR | The manuscript doesn't report the sequence generation method. |
|  | Allocation concealment? | UNCLEAR | No method reported for allocation concealment. |
|  | Blinding of participants and personnel? | UNCLEAR | There aren't sufficient information to permit judgement. |
|  | Blinding of outcome assessment? | UNCLEAR | There aren't sufficient information to permit judgement. |
|  | Complete outcome data addressed? | YES | No patients lost after randomization. |
|  | Free of selective reporting? | UNCLEAR | There aren't sufficient information to permit judgement. |
|  | Free of other bias? | YES | There is no evidence of other bias. |
|  | OVERALL RISK OF BIAS | MODERATE |  |
| Lucchinetti E 2012 | Adequate sequence generation? | YES | Quote: "randomization (block size 10) with no further stratification was generated by an independent person using a computer random number generator" |
|  | Allocation concealment? | YES | Quote: " numbered, sealed, opaque envelopes" |
|  | Blinding of participants and personnel? | UNCLEAR | There aren't sufficient information to permit judgement. |
|  | Blinding of outcome assessment? | YES | Quote: "Collection and analyses of all clinical and laboratory data were performed by study personnel blinded for group assignment" |
|  | Complete outcome data addressed? | YES | No patients lost after randomization. |
|  | Free of selective reporting? | YES | Outcomes have been reported in the pre-specified way and the trial was registered with ClinicalTrials.gov and issued with the identification number NCT00546390. |
|  | Free of other bias? | YES | There is no evidence of other bias. |
|  | OVERALL RISK OF BIAS | LOW |  |
| Meybohm P 2013 | Adequate sequence generation? | UNCLEAR | The manuscript doesn't report the sequence generation method. |
|  | Allocation concealment? | YES | Quote: "Sealed envelopes were used for randomization" |
|  | Blinding of participants and personnel? | YES | Quote: "This study is a prospective randomized double-blind" |
|  | Blinding of outcome assessment? | UNCLEAR | There aren't sufficient information to permit judgement. |
|  | Complete outcome data addressed? | NO | Data were not analyzed according to the intention-to-treat principle. |
|  | Free of selective reporting? | UNCLEAR | There aren't sufficient information to permit judgement. |
|  | Free of other bias? | YES | There is no evidence of other bias. |
|  | OVERALL RISK OF BIAS | MODERATE |  |
| Meco M 2007 | Adequate sequence generation? | YES | Quote: "The randomisation management was delegated to a person unconnected to the clinical experimentation." |
|  | Allocation concealment? | YES | Quote: "Patients were randomly divided by opening of a sealed envelope the evening before the surgical procedure" |
|  | Blinding of participants and personnel? | YES | Quote: "No operator involved in the care of the patient in every phase had any knowledge of the group to which each single patient belonged" |
|  | Blinding of outcome assessment? | NO | Quote:"The person who collected the data and the individual who carried out the statistical analysis had knowledge of the group to which each single patient belonged" |
|  | Complete outcome data addressed? | YES | No patients lost after randomization. |
|  | Free of selective reporting? | UNCLEAR | There aren't sufficient information to permit judgement. |
|  | Free of other bias? | YES | There is no evidence of other bias. |
|  | OVERALL RISK OF BIAS | LOW |  |
| Musialowicz T 2007 | Adequate sequence generation? | UNCLEAR | The manuscript doesn't report the sequence generation method. |
|  | Allocation concealment? | UNCLEAR | No method reported for allocation concealment. |
|  | Blinding of participants and personnel? | UNCLEAR | There aren't sufficient information to permit judgement. |
|  | Blinding of outcome assessment? | YES | Quote: "A trained physicist, who was blinded to the group allocation of patients, performed the MLAEPs data analyses" |
|  | Complete outcome data addressed? | UNCLEAR | There aren't sufficient information to permit judgement. |
|  | Free of selective reporting? | UNCLEAR | There aren't sufficient information to permit judgement. |
|  | Free of other bias? | NO | Quote: "One of the test variables, BIS, was used as a clinical end-point to achieve a standardized depth of anaesthesia, and thereby ideally it should not differ between the groups at any time point. This appears to give an unintentional bias against AEP" |
|  | OVERALL RISK OF BIAS | MODERATE |  |
| Rahman IA 2010 | Adequate sequence generation? | YES | Quote: "computer-generated randomization schedules" |
|  | Allocation concealment? | YES | Quote: "numbered sealed envelopes" |
|  | Blinding of participants and personnel? | YES | Quote: "Patients, investigators, anesthetists, surgeons, echocardiographers, and critical care teams were all blinded to group allocation" |
|  | Blinding of outcome assessment? | UNCLEAR | There aren't sufficient information to permit judgement. |
|  | Complete outcome data addressed? | YES | No patients lost after randomization. |
|  | Free of selective reporting? | UNCLEAR | There aren't sufficient information to permit judgement. |
|  | Free of other bias? | NO | Quote: "Subtle differences in conditioning status may occur in patients presenting with urgent coronary syndrome status that prevents further conditioning in this group" |
|  | OVERALL RISK OF BIAS | LOW |  |
| Royse CF 2011 | Adequate sequence generation? | YES | Quote: "The allocation sequence was generated by computer random number generation" |
|  | Allocation concealment? | YES | Quote: "allocation was placed in sequentially numbered opaque sealed envelopes by a non-investigator" |
|  | Blinding of participants and personnel? | YES | Quote: "The treating clinicians were not blinded to the assignment group, but all other staff involved in both the collection and collation of data, and administration of neurocognitive testing, were blinded to group allocation, participants were not informed of assignment" |
|  | Blinding of outcome assessment? | YES | Quote: "A blinded interim analysis was performed on the primary endpoint" |
|  | Complete outcome data addressed? | YES | Quote: "Intention to treat analysis was performed" |
|  | Free of selective reporting? | UNCLEAR | There aren't sufficient information to permit judgement. |
|  | Free of other bias? | NO | Quote: "Participants were not informed of assignment, but may have detected the smell of gaseous induction of anaesthesia in the desflurane group and therefore it cannot be assured that they were blinded" |
|  | OVERALL RISK OF BIAS | LOW |  |
| Saxena P 2013 | Adequate sequence generation? | UNCLEAR | The manuscript doesn't report the sequence generation method. |
|  | Allocation concealment? | UNCLEAR | No method reported for allocation concealment. |
|  | Blinding of participants and personnel? | YES | Quote: "This was a randomized, double-blind controlled prospective trial" |
|  | Blinding of outcome assessment? | UNCLEAR | There aren't sufficient information to permit judgement |
|  | Complete outcome data addressed? | YES | No patients lost after randomization. |
|  | Free of selective reporting? | UNCLEAR | There aren't sufficient information to permit judgement. |
|  | Free of other bias? | YES | There is no evidence of other bias. |
|  | OVERALL RISK OF BIAS | LOW |  |
| Schoen J 2011 | Adequate sequence generation? | UNCLEAR | The manuscript doesn't report the sequence generation method. |
|  | Allocation concealment? | UNCLEAR | No method reported for allocation concealment. |
|  | Blinding of participants and personnel? | YES | Quote: "The investigators were blinded to the anaesthesia protocol" |
|  | Blinding of outcome assessment? | UNCLEAR | There aren't sufficient information to permit judgement. |
|  | Complete outcome data addressed? | NO | Quote: "Sixty-four patients were randomized for each anaesthesia regimen; 11 patients could not be analysed for various reasons" |
|  | Free of selective reporting? | UNCLEAR | There aren't sufficient information to permit judgement even if the study is registered in the European Clinical Trials Database no. 2005-004928-39 and the ISRCTN Register (ISRCTN44821042) |
|  | Free of other bias? | NO | Quote: " the effective group size was much smaller … This certainly weakens the information given by the present study" |
|  | OVERALL RISK OF BIAS | MODERATE |  |
| Soro S 2012 | Adequate sequence generation? | YES | Quote: "Simple randomisation was carried out using a random number table generator" |
|  | Allocation concealment? | YES | Quote: "Numbers were distributed in sealed, opaque envelopes which were opened at the beginning of anaesthesia" |
|  | Blinding of participants and personnel? | YES | Quote: "In order to prevent any bias, a strict double-blind, doubledummy design was used" |
|  | Blinding of outcome assessment? | UNCLEAR | There aren't sufficient information to permit judgement. |
|  | Complete outcome data addressed? | NO | Two patients lost in Propofol group after randomization. |
|  | Free of selective reporting? | UNCLEAR | There aren't sufficient information to permit judgement. |
|  | Free of other bias? | YES | There is no evidence of other bias. |
|  | OVERALL RISK OF BIAS | LOW |  |
| Story DA 2001 | Adequate sequence generation? | YES | Quote: "table of random numbers to allocate patients" |
|  | Allocation concealment? | YES | Quote: "numbered envelopes" |
|  | Blinding of participants and personnel? | YES | Quote: "Intensive care unit staff and patients were blinded to the drug allocation" |
|  | Blinding of outcome assessment? | UNCLEAR | There aren't sufficient information to permit judgement. |
|  | Complete outcome data addressed? | YES | Quote: "Analysis was performed on an intention-to-treat basis." |
|  | Free of selective reporting? | UNCLEAR | There aren't sufficient information to permit judgement. |
|  | Free of other bias? | NO | Quote: "None of the researchers for this study were paid by drug companies. However, we received equivalent funding from the suppliers of propofol (AstraZeneca) and the suppliers of isoflurane and sevoflurane (Abbott Australasia)." |
|  | OVERALL RISK OF BIAS | LOW |  |
| Tempe DK 2011 | Adequate sequence generation? | UNCLEAR | The manuscript doesn't report the sequence generation method. |
|  | Allocation concealment? | YES | Quote: "Patients were randomly (by opening of an envelope)" |
|  | Blinding of participants and personnel? | YES | Quote: " After surgery, patients were transferred to the intensive care unit and managed as per the standard protocol by a team who was blinded to the anesthetic regimen used" |
|  | Blinding of outcome assessment? | UNCLEAR | There aren't sufficient information to permit judgement. |
|  | Complete outcome data addressed? | NO | Quote: "Five patients (3 from the propofol group and 2 from the isoflurane group) were excluded from the study due to severe hemodynamic instability" |
|  | Free of selective reporting? | UNCLEAR | There aren't sufficient information to permit judgement. |
|  | Free of other bias? | NO | Quote: "the concentration of isoflurane, dose of propofol, and duration of their use was not uniform in all patients. Thus, dose dependency of their cardioprotective effects was uncertain" |
|  | OVERALL RISK OF BIAS | MODERATE |  |
| Thielmann M 2010 | Adequate sequence generation? | UNCLEAR | The manuscript doesn't report the sequence generation method. |
|  | Allocation concealment? | UNCLEAR | No method reported for allocation concealment. |
|  | Blinding of participants and personnel? | NO | Quote: "Patients, cardiac surgeons, as well as the intensive care staff were blind to treatment allocation, whereas the anesthesiologists who applied the RIPC protocol were not blind" |
|  | Blinding of outcome assessment? | UNCLEAR | There aren't sufficient information to permit judgement. |
|  | Complete outcome data addressed? | NO | Quote: "After randomization, three more patients had to be excluded" |
|  | Free of selective reporting? | UNCLEAR | There aren't sufficient information to permit judgement even if the study was approved by the Essen University Hospital Ethics Committee (Ref#, 08- 3683). |
|  | Free of other bias? | YES | There is no evidence of other bias. |
|  | OVERALL RISK OF BIAS | MODERATE |  |
| Thielmann M 2013 | Adequate sequence generation? | YES | Quote: "Codes were computer generated and kept in sealed envelopes at a central location." |
|  | Allocation concealment? | YES | Quote: "Codes were computer generated and kept in sealed envelopes at a central location." |
|  | Blinding of participants and personnel? | YES | Quote: "Patients, cardiac surgeons, and intensive-care physicians were unaware of treatment assignment" |
|  | Blinding of outcome assessment? | UNCLEAR | There aren't sufficient information to permit judgement. |
|  | Complete outcome data addressed? | YES | Quote: "Data of all randomised patients were included in the effi cacy analyses, according to the intention-to-treat principle" |
|  | Free of selective reporting? | UNCLEAR | There aren't sufficient information to permit judgement. |
|  | Free of other bias? | YES | There is no evidence of other bias. |
|  | OVERALL RISK OF BIAS | LOW |  |
| Tritapepe L 2003 | Adequate sequence generation? | UNCLEAR | The manuscript doesn't report the sequence generation method |
|  | Allocation concealment? | UNCLEAR | No method reported for allocation concealment |
|  | Blinding of participants and personnel? | UNCLEAR | There aren't sufficient information to permit judgement |
|  | Blinding of outcome assessment? | UNCLEAR | There aren't sufficient information to permit judgement |
|  | Complete outcome data addressed? | YES | No patients lost after randomization |
|  | Free of selective reporting? | UNCLEAR | There aren't sufficient information to permit judgement |
|  | Free of other bias? | YES | There is no evidence of other bias |
|  | OVERALL RISK OF BIAS | MODERATE |  |
| Tritapepe L 2007 | Adequate sequence generation? | YES | Quote: "The details of the randomization, created by a computer generated list in blocks of 10, were …" |
|  | Allocation concealment? | YES | Quote: "… were contained in a set of sealed envelops that were opened at the start of naesthesia." |
|  | Blinding of participants and personnel? | YES | Quote: "All study personnel and participants were blinded to treatment assignment" |
|  | Blinding of outcome assessment? | NO | Quote: "All study personnel and participants were blinded to treatment assignment for the duration of the study with the exception of the cardiac anaesthesiologists, who were not involved in data collection, data entry or data analysis" |
|  | Complete outcome data addressed? | YES | Quote: "All data were analysed according to the intention to-treat principle" |
|  | Free of selective reporting? | UNCLEAR | There aren't sufficient information to permit judgement. |
|  | Free of other bias? | YES | There is no evidence of other bias |
|  | OVERALL RISK OF BIAS | LOW |  |
| Wagner R 2010 | Adequate sequence generation? | YES | Quote: "A computer-generated table of random numbers was used for randomization" |
|  | Allocation concealment? | UNCLEAR | No method reported for allocation concealment. |
|  | Blinding of participants and personnel? | YES | Quote: The surgery team, evaluators and data analysts were blinded to group assignment" |
|  | Blinding of outcome assessment? | YES | Quote: The surgery team, evaluators and data analysts were blinded to group assignment" |
|  | Complete outcome data addressed? | NO | Quote: "The analysis was per-protocol" |
|  | Free of selective reporting? | UNCLEAR | There aren't sufficient information to permit judgement. |
|  | Free of other bias? | YES | There is no evidence of other bias. |
|  | OVERALL RISK OF BIAS | LOW |  |
| Williams JM 2012 | Adequate sequence generation? | YES | Quote: "… using an online randomisation sequence generator" |
|  | Allocation concealment? | YES | Quote: "Treatment group allocation was concealed in sequentially numbered opaque envelopes" |
|  | Blinding of participants and personnel? | YES | Quote: "We completed a double-blind, randomised, controlled trial" |
|  | Blinding of outcome assessment? | UNCLEAR | There aren't sufficient information to permit judgement. |
|  | Complete outcome data addressed? | NO | Quote: "Excluded from analysis due to use of immunosuppressant (n=1) |
|  | Free of selective reporting? | UNCLEAR | There aren't sufficent information to permit judgement even if the study was registered on the Australian New Zealand Clinical Trials Registry ACTRN 12609000965202. |
|  | Free of other bias? | YES | There is no evidence of other bias. |
|  | OVERALL RISK OF BIAS | LOW |  |
| Wu Q 2011 | Adequate sequence generation? | UNCLEAR | The manuscript doesn't report the sequence generation method. |
|  | Allocation concealment? | UNCLEAR | No method reported for allocation concealment. |
|  | Blinding of participants and personnel? | UNCLEAR | There aren't sufficient information to permit judgement. |
|  | Blinding of outcome assessment? | UNCLEAR | There aren't sufficient information to permit judgement. |
|  | Complete outcome data addressed? | YES | No patients lost after randomization |
|  | Free of selective reporting? | UNCLEAR | There aren't sufficient information to permit judgement. |
|  | Free of other bias? | YES | There is no evidence of other bias. |
|  | OVERALL RISK OF BIAS | MODERATE |  |
| Xie J 2012 | Adequate sequence generation? | YES | Quote: "patients were randomly assigned to either the RIPC group or the control group using a random numbers table" |
|  | Allocation concealment? | UNCLEAR | No method reported for allocation concealment |
|  | Blinding of participants and personnel? | YES | Quote: "The patients, ultrasound physicians, and individuals detecting the samples and analysing the data were all blinded" |
|  | Blinding of outcome assessment? | YES | Quote: "The patients, ultrasound physicians, and individuals detecting the samples and analysing the data were all blinded" |
|  | Complete outcome data addressed? | YES | Quote: "The analysis was by intention to treat" |
|  | Free of selective reporting? | UNCLEAR | There aren't sufficient information to permit judgement |
|  | Free of other bias? | YES | There is no evidence of other bias |
|  | OVERALL RISK OF BIAS | LOW |  |
| Yildirim V 2009 | Adequate sequence generation? | YES | Quote: "A computer-generated random code was used" |
|  | Allocation concealment? | YES | Quote: "Subjects were assigned the treatment numbers in ascending chronological order of admission in the study. The participant randomization assignment was concealed in an envelope until the start of anesthesia" |
|  | Blinding of participants and personnel? | YES | Quote: "The surgeons, research assistants, and medical and nursing staff in the intensive care unit (ICU) and on the ward were blinded to the group assignments" |
|  | Blinding of outcome assessment? | UNCLEAR | There aren't sufficient information to permit judgement |
|  | Complete outcome data addressed? | YES | No patients lost after randomization |
|  | Free of selective reporting? | UNCLEAR | There aren't sufficient information to permit judgement |
|  | Free of other bias? | YES | There is no evidence of other bias |
|  | OVERALL RISK OF BIAS | LOW |  |
| Young PJ 2012 | Adequate sequence generation? | YES | Quote: "The random allocation sequence was generated by a third party using an online randomisation sequence generator (http://www.randomization.com) using block andomisation with a block size of eight." |
|  | Allocation concealment? | YES | Quote: "The allocation to RIPC or control was concealed in sequentially numbered opaque envelopes" |
|  | Blinding of participants and personnel? | YES | Quote: "We conducted a double blind, parallel-groups, phase 2b randomised controlled trial" |
|  | Blinding of outcome assessment? | UNCLEAR | There aren't sufficient information to permit judgement |
|  | Complete outcome data addressed? | YES | Data were analyzed according to the intention-to-treat principle. |
|  | Free of selective reporting? | UNCLEAR | There aren't sufficient information to permit judgement |
|  | Free of other bias? | YES | There is no evidence of other bias |
|  | OVERALL RISK OF BIAS | LOW |  |
| Ziemmerman 2011 | Adequate sequence generation? | UNCLEAR | The manuscript doesn't report the sequence generation method. Quote: "… generated by the study coordinator." |
|  | Allocation concealment? | YES | Quote: "opaque, sealed envelope containing the patient’s group assignment" |
|  | Blinding of participants and personnel? | YES | Quote:"Only patients and those performing creatinine and NGAL assays were blinded to randomization." |
|  | Blinding of outcome assessment? | NO | Quote: "Outcomes assessors were not blinded to group assignment." |
|  | Complete outcome data addressed? | YES | Quote: "For the primary analysis, we analyzed all end points on an intention-to-treat basis" |
|  | Free of selective reporting? | UNCLEAR | There aren't sufficient information to permit judgement |
|  | Free of other bias? | YES | There is no evidence of other bias |
|  | OVERALL RISK OF BIAS | LOW |  |
